# Phosphite synthetic auxotrophy as an effective biocontainment strategy for the industrial chassis *Pseudomonas putida*

**DOI:** 10.1186/s12934-022-01883-5

**Published:** 2022-08-08

**Authors:** Enrique Asin-Garcia, Christos Batianis, Yunsong Li, James D. Fawcett, Ivar de Jong, Vitor A. P. Martins dos Santos

**Affiliations:** 1 Laboratory of Systems and Synthetic Biology, Wageningen University & Research, Wageningen, 6708 WE The Netherlands; 2 LifeGlimmer GmbH, 12163 Berlin, Germany; 3 Bioprocess Engineering Group, Wageningen University & Research, Wageningen, 6700 AA The Netherlands; 4 Department of Life Sciences, Imperial College London, Exhibition Road, South Kensington, London, SW72BX UK; 5 The Novo Nordisk Foundation Center for Biosustainability, Technical University of Denmark, 2800 Kgs. Lyngby, Denmark

**Keywords:** *Pseudomonas putida*, Biocontainment, Synthetic auxotrophy, Phosphite, Non-sterile conditions, NADH

## Abstract

The inclusion of biosafety strategies into strain engineering pipelines is crucial for *safe-by-design* biobased processes. This in turn might enable a more rapid regulatory acceptance of bioengineered organisms in both industrial and environmental applications. For this reason, we equipped the industrially relevant microbial chassis *Pseudomonas putida* KT2440 with an effective biocontainment strategy based on a synthetic dependency on phosphite, which is generally not readily available in the environment. The produced PSAG-9 strain was first engineered to assimilate phosphite through the genome-integration of a phosphite dehydrogenase and a phosphite-specific transport complex. Subsequently, to deter the strain from growing on naturally assimilated phosphate, all native genes related to its transport were identified and deleted generating a strain unable to grow on media containing any phosphorous source other than phosphite. PSAG-9 exhibited fitness levels with phosphite similar to those of the wild type with phosphate, and low levels of escape frequency. Beyond biosafety, this strategy endowed *P. putida* with the capacity to be cultured under non-sterile conditions using phosphite as the sole phosphorous source with a reduced risk of contamination by other microbes, while displaying enhanced NADH regenerative capacity. These industrially beneficial features complement the metabolic advantages for which this species is known for, thereby strengthening it as a synthetic biology chassis with potential uses in industry, with suitability towards environmental release.

**Supplementary Information:**

The online version contains supplementary material available at 10.1186/s12934-022-01883-5.

## Introduction

### Safe-by-design industrial chassis

The KT2440 strain of the gram-negative soil bacterium *Pseudomonas putida* has high potential as a host for the development of enabling synthetic biology technologies, and as a metabolically versatile chassis with a proven safety record and a tolerance towards genome reduction, toxic compounds and redox stressors [1–4]. With an increasingly accurate annotated genome [5] and a diverse collection of in silico and genome modification tools [6], the strain is well suited for high-throughput and automated engineering through the performance of iterative Design-Build-Test-Learn (DBTL) cycles in the context of biofoundries, biomanufacturing and industrial biotechnology in general [7, 8]. Thus, the generation of engineered *P. putida* strains with defined and beneficial industrial characteristics could play an enabling role in standardizing this microbe as a chassis for future generation of synthetic biology-derived products and for its use in potential environmental applications [9].

As a consequence of its increasing popularity in research and industry, *P. putida* is undergoing ever more profound genetic and metabolic engineering [10, 11]. This has led to a growing focus on improving the biosafety credentials of the engineered strains to prevent their escape from closed settings and their uncontrolled transmission in open or semi-open environments [12–14]. To this end, advocates of biosafety believe that the implementation of a safe-by-design approach would be necessary to facilitate commonplace use of heavily engineered strains for applications involving environmental release [15, 16]. However, genetic safeguard technologies, still in their infancy for real-world applications, are not widely used within industry [17]. With biomanufacturing becoming a driving force behind the development and regulation of engineered organisms, industries will likely be required to consider biosafety upfront and incorporate biocontainment strategies in the early phases of strain development. In keeping with the synthetic biology tenets of standardisation and modularity, it would be beneficial if the biocontainment strategies selected for this purpose were versatile and portable between different synthetic biology chassis [18]. “Plug-in” biocontainment modules would be indispensable in the rapid construction of strains with built-in biosafety, which could help in streamlining the process of regulation [19].

### Phosphite synthetic auxotrophy

**Fig. 1:**
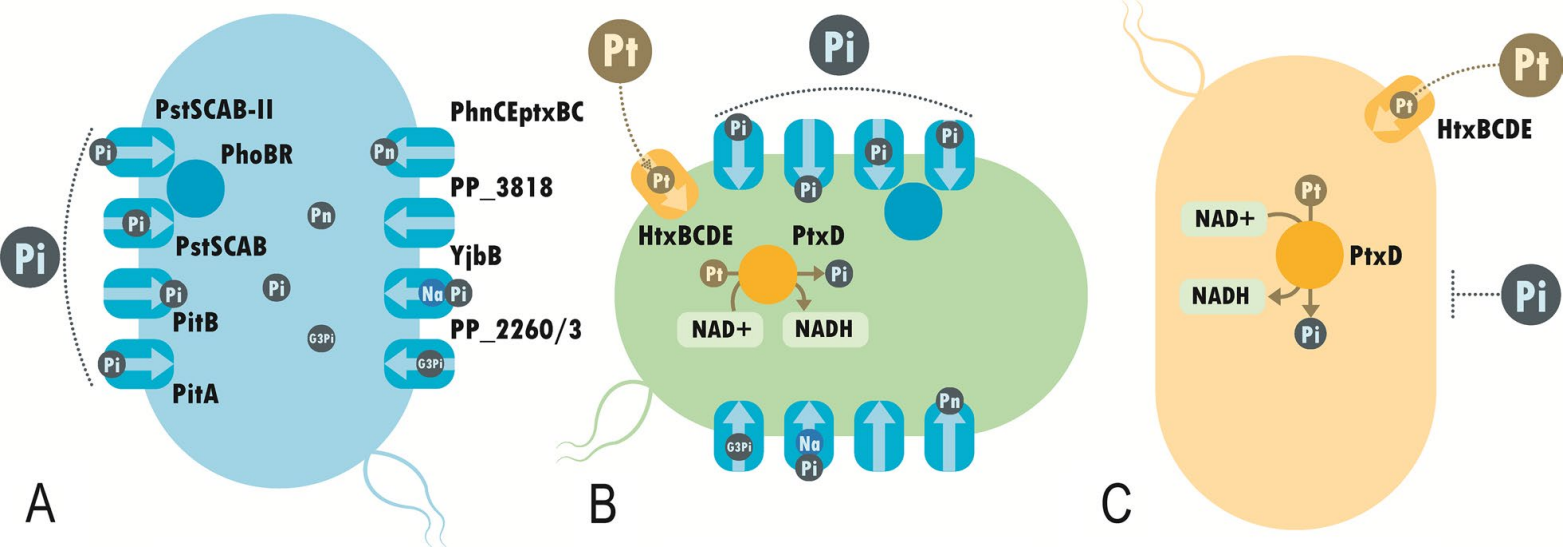
Graphical representation of the different stages of the generation of a phosphite auxotrophic *P. putida* strain. **A** In blue, *P. putida* KT2440 with its native Pi-transport-related genes. Pi, Pn and G3Pi stand for phosphite, phosphonate and glycerol-3-phosphate. **B** In green, *P. putida* PSAG with its corresponding native genes, plus the hypophosphite transporter HtxBCDE from *P. stutzeri* WM88 and the phosphite dehydrogenase PtxD from *Ralstonia sp.* 4506. Pt stands for phosphite. **C** In yellow, *P. putida* PSAG-9 equipped only with the phosphite assimilation genes *htxBCDE* and *ptxD* and deprived of its native Pi-transport related genes

With a non-complex technical implementation and a current record for the lowest detectable escape frequency of any genetic safeguard, synthetic auxotrophy is a good candidate to examine the potential portability of biocontainment traits among chassis [13, 20–22]. One of the most recent and successful strategies is the alteration of the phosphorous (P) metabolism to engineer a synthetic auxotrophy as an effective means of biocontainment. This auxotrophy has so far been successfully incorporated in both *E. coli* and the cyanobacteria *Synechococcus elongatus* PCC 7942, two gram-negative model organisms that are representative of effective synthetic biology chassis [23, 24]. Phosphate (Pi, PO_4_^3−^), the biologically available form of P, is utilised in several key biological molecules, including nucleic acids, phospholipids and ATP. Most bacteria possess multiple Pi transporter genes [25] to provide transporter redundancy, in the off chance that one acquires a deleterious mutation, and also allows for the fine tuning of Pi import through differential gene regulation. The genome of *P. putida* KT2440 [5] indicates the presence of two low-affinity inorganic Pi transporters, PitB and PitA, and, unlike in *E. coli*, two copies of the high-affinity inorganic Pi transport complex, PstSCAB and PstSCAB-II (Fig. 1A). Additionally, orthologs of other genes that have previously been related to phosphate transport in *E. coli* can also be found in the KT2440 genome, including the phosphonate transporter PhnCEptxBC and the organic Pi transporter for glycerol-3-phosphate (G3Pi) encoded by the operon *PP_2260/3* [26] (Fig. 1A).

Phosphite (Pt, HPO_3_^2−^), a reduced form of Pi, is an uncommon form of P in the environment and occurs naturally at very low concentrations [27–29]. Nevertheless, several species of bacteria have been evolutionary programmed to metabolise Pt (in this article Pt stands for phosphite; do not confuse with the element Platinum, also abbreviated as Pt in other contexts) and utilise it as a source of P. The genes that comprise these metabolic processes can be isolated and used to endow a Pt-assimilation behaviour in a recombinant organism. In previous studies, the researchers inserted the Pt specific ABC transporter HtxBCDE, derived from the *Pseudomonas stutzeri* strain WM88, and the Pt dehydrogenase PtxD, taken from *Ralstonia* sp. 4506, to convert the newly imported Pt into Pi [23, 24, 30, 31]. Once the Pt utilization pathway was constructed, the ability of the engineered strain to incorporate the metabolizable Pi needed to be eliminated to achieve a full auxotrophy.

Here, we established a Pt auxotrophy in *P. putida* KT2440 by integrating a functional Pt assimilation pathway and by deleting all the relevant native Pi transporters in order to produce a strain capable of utilising Pt, but not Pi, as the sole P source. In addition, the characteristics of this strategy equipped the strain with other properties, beneficial for industrial applications beyond biosafety, including a competitive growth advantage over contaminating organisms on media containing Pt as the sole P source. Thus, the development of this biocontainment strategy positions *P. putida* as a safer biological chassis with potential uses in research and industrial biotechnology, especially towards environmental applications.

## Results

### The *htxBCDE* and *ptxD* genes permit *P. putida* to utilise Pt as a P source

**Fig. 2:**
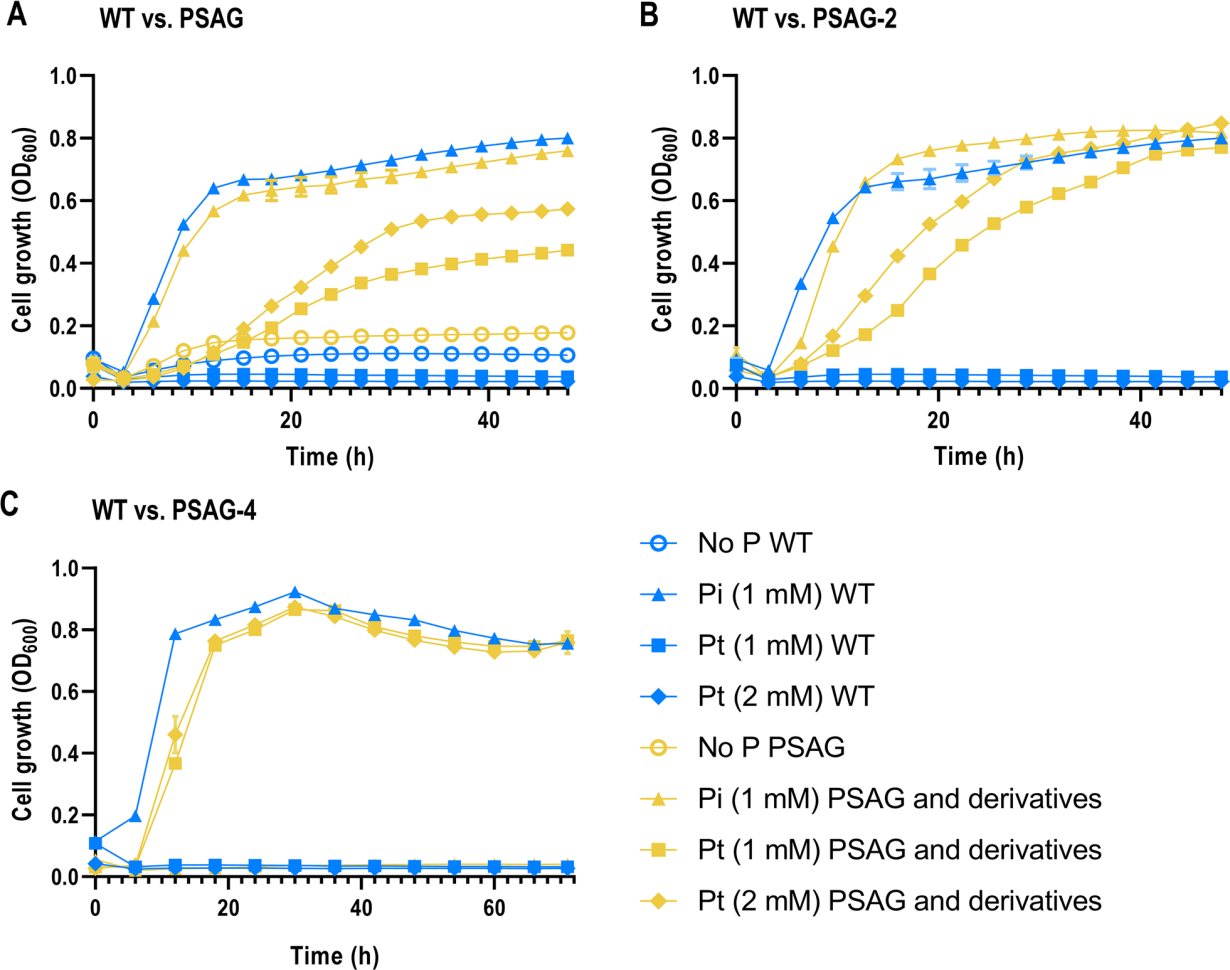
Growth of *P. putida* in different P sources before and after addition of the PSAG assimilation genes and the removal of its Pi native transporters. **A** Growth of *P. putida* wild type (blue) and PSAG (yellow) in MOPS with different P sources. **B** Growth of *P. putida* wild type (blue) and PSAG-2 (yellow) in MOPS with different P sources. **C** Growth of *P. putida* wild type (blue) and PSAG-4 (yellow) in MOPS with different P sources. Growth was monitored by measuring OD_600_ in an ELx808 Absorbance Microplate Reader (BioTek Instruments, Inc., VT, U.S.). Error bars represent the standard deviation among biological duplicates and technical triplicates for each condition

To confirm that *P. putida* KT2440 cannot naturally utilise and grow on Pt as a P source, a wild type *P. putida* strain was first seeded into MOPS minimal medium containing 50 mM of glucose and relevant P sources (indicated in Fig. 2A). As expected, the strain was able to grow in media supplemented with inorganic Pi at a concentration of 1 mM. This data also suggests that *P. putida* KT2440 is unable to grow without any P source or under Pt conditions, confirming that Pt cannot be utilized as a P source. A negligible growth was observed in the absence of P source, likely supported by internally accumulated Pi [32].

As expected from previous studies [23, 24], the introduction of the hypophosphite transporter HtxBCDE, derived from *P. stutzeri* WM88, and the phosphate dehydrogenase PtxD from *Ralstonia sp.* 4506 (in this study collectively referred to as the phosphite synthetic auxotrophy genes, abbreviated as PSAG) into the wild type KT2440 resulted in the strain gaining the ability to grow on Pt (Fig. 2A). After integrating both the *htxBCDE* operon and the *ptxD* gene under the control of the lac and tac promoters, respectively, into the *att*Tn7 site of KT2440 genome, the growth of the PSAG strain on Pt provided evidence that the PSAG were functionally expressed and metabolically active. *P. putida* PSAG (Fig. 1B) was able to grow on Pt to a cell density close to that of the wild type on Pi containing media, although the latter showed a shorter lag phase, 243% higher growth rate and achieved a 39.5% higher final cell density after 48 h than PSAG (Fig. 2A). *P. putida* PSAG also appeared to grow on Pt at both the tested concentrations of 1 and 2 mM. Growth was significantly higher in the 2 mM Pt condition (25.9% higher growth rate and 29.8% higher final OD_600_), indicating a growth-rate dependency on ambient Pt concentration. However, growth in 2 mM Pt was still significantly lower than growth in Pi at 1 mM.

Although there appears to be no native specific Pt transporters in *P. putida*, it was expected that the cells were capable of importing Pt through the native Pi transporters due to the structural similarities between the two forms of P [23]. To confirm this, a 24-h growth assay was prepared to examine if a *P. putida* strain containing *ptxD* but no *htxBCDE* in its genome was able to grow on a Pt source (Additional file 1: Figure S1). The PtxD-containing strain could indeed grow on Pt, indicating that Pt is able to enter the cell most likely through the native Pi transporters. This strain showed a significantly lower final OD_600_ than the *P. putida* PSAG on Pt (12.2% lower than PSAG), suggesting that the HtxBCDE transporter does function in importing Pt, and is more efficient at Pt transport than any native Pi transporter. On a different note, the fact that the native Pi transporters can incorporate Pt while the wild type KT2440 is unable to grow with Pt as sole P source suggests that no native *P. putida* enzyme can convert Pt to Pi in an efficient enough manner as to provide sufficient levels of Pi for the cell to grow. This is relevant due to the existence of a native PtxD from *P. putida* encoded by *PP_3376*, which was inferred from homology. Since the native PtxD does not seem to mediate our function of interest, we do not consider it in this study and refer to the *Ralstonia* sp. 4506’s protein when we mention PtxD, unless stated otherwise.

### Deletion of *P. putida*’s native Pi transporters prevents growth on Pi and enhances growth on Pt

Once we verified that the introduction of *htxBCDE* and *ptxD* allowed *P. putida* PSAG to grow on Pt as sole P source, the strain was engineered to eliminate its ability of growing on Pi. This process was initiated by deleting the low affinity inorganic Pi transporters PitB and PitA, resulting in strains PSAG-1 (Δ*pitB*) and PSAG-2 (Δ*pitB* Δ*pitA*). Their removal appeared to show no major growth deficit compared to the wild type strain when growing on Pi (Fig. 2B and Additional file 1: Figure S2A), likely because of the high affinity transporters entirely taking over the Pi transport. Unexpectedly, PSAG-1 and PSAG-2 showed a considerable increase in growth on Pt (Fig. 2B and Additional file 1: Figure S2A) compared to its parental strain *P. putida* PSAG (Fig. 2A). We initially hypothesized that the subunits of the native and heterologous transporters could mix and interfere with each other. However, the components of HtxBCDE share homology with Phn-like proteins [28, 33] instead of the Pit transporters, which made us discard this option. Alternatively, this phenomenon could be explained by the lack of competition for membrane space that would be present in the strains expressing both the HtxBCDE and the Pit transporters, allowing more Pt-specific transporters to be present at the cell surface. Membrane space competition can be expected from high transporter expression, which is here granted by the strong lac promoter controlling HtxBCDE. On 2 mM of Pt, *P. putida* PSAG-2 meets the final OD of the same strain grown on 1 mM of Pi after 48 h, establishing the suitability of this Pt concentration in the culture of the auxotroph and suggesting that the Pt concentration used is not yet limiting (Fig. 2B).

Following the removal of the low affinity Pi transporters PitB and PitA, the high-affinity PstSCAB and PstSCAB-II Pi transporters were deleted, resulting in strains PSAG-3 (Δ*pitB* Δ*pitA* Δ*pstSCAB*) and PSAG-4 (Δ*pitB* Δ*pitA* Δ*pstSCAB* Δ*pstSCAB-II*) (Fig. 2C and Additional file 1: Figure S2B). As both gene clusters are upregulated during Pi starvation [34], their removal would be likely to significantly hinder the ability of the cell to import inorganic Pi. As expected, the removal of both transporters was sufficient to completely supress growth in minimal Pi media for at least 72 h (Fig. 2C). When grown in either 1 or 2 mM of Pt, *P. putida* PSAG-4 reached an OD comparable to that of the wild type in 1 mM Pi but after a slightly longer lag phase (Fig. 2C). This 4-knock-out strain in Pt achieved the closest growth profile to the wild type grown in Pi, with a shortly elongated lag phase but comparable growth rate.

While *P. putida* PSAG-4 was not able to grow in MOPS-Pi for 72 h, it did in rich media such as LB, probably due to the presence of alternative P sources (likely contained in the yeast extract) or the cotransport of Pi when associated to other molecules missing in the minimal medium. To prevent PSAG-4 from importing any other form of P other than Pt, we knocked-out a series of genes that were involved in P transport. First, we deleted the operon *phnCEptxBC*, whose product is mainly involved in metabolism and transport of phosphonates [28, 35]. Secondly, the *PP_3818* gene, which had been predicted to encode a periplasmic phosphate-binding protein [36], was subsequently deleted. These two strains, called PSAG-5 (Δ*pitB* Δ*pitA* Δ*pstSCAB* Δ*pstSCAB-II* Δ*phnCEptxBC*) and PSAG-6 (Δ*pitB* Δ*pitA* Δ*pstSCAB* Δ*pstSCAB-II* Δ*phnCEptxBC* Δ*PP_3818*), respectively, were still able to grow in LB without Pt (not shown). While the former showed no differences in final OD when compared to the wild type in MOPS medium as it had happened with the previous mutant PSAG-4, the latter started showing lower final OD levels in MOPS-Pt than those of the wild type strain in MOPS-Pi. Furthermore, the lag phases of these two mutants kept being slightly longer than the wild type controls (Additional file 1: Figure S2C and D). Following, the putative glycerol-phosphate transporter encoded by *PP_2260/3* was removed to avoid the incorporation of trace amounts of Pi via organic Pi transporters. Lastly, the Na^+^/Pi-symporter YjbB was eliminated to exclude the possibility of Pi transport into the cell in Na^+^-rich conditions, even though previous studies indicated that YjbB has none or little Pi-uptake activity and is exclusively dedicated to Pi-export in *E. coli* [37]. The growth of these two strains, PSAG-7 (Δ*pitB* Δ*pitA* Δ*pstSCAB* Δ*pstSCAB-II* Δ*phnCEptxBC* Δ*PP_3818* Δ*PP_2260/3*) and PSAG-8 (Δ*pitB* Δ*pitA* Δ*pstSCAB* Δ*pstSCAB-II* Δ*phnCEptxBC* Δ*PP_3818* Δ*PP_2260/3* Δ*yjbB*), was monitored in both LB and LB-Pt, showing no differences in growth rate, lag phase and final OD between the two conditions. However, while the lag phase was the same, growth rate and final OD of these two mutants were lower than those of the wild type in the same media (Additional file 1: Figure S2E, F).

Furthermore, *P. putida* contains a complete set of *xcp* genes (type II secretion system) that participates in the secretion of enzymes involved in phosphate acquisition under low Pi conditions [38]. Overexpression of the corresponding genes is controlled by a two-component regulatory system that consists of PhoB as the response regulator and PhoR as the histidine kinase [39]. Given the high number of phosphatases and other enzymes involved in this Pi scavenging process, we opted for the removal of the *phoBR* regulon to avoid the activation of the system under Pi-limiting growth conditions, which resulted in *P. putida* PSAG-9 (Fig. 1C).

**Fig. 3:**
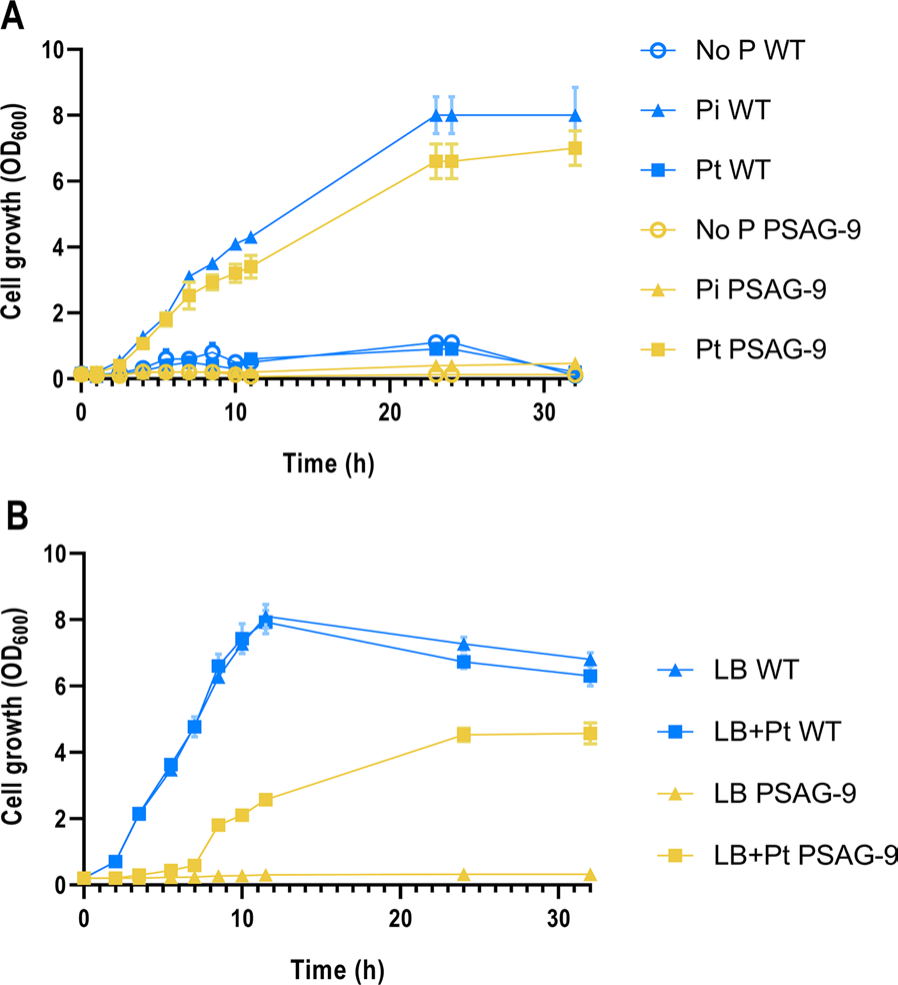
PSAG-9 strain only grows on Pt-supplemented media. **A** Growth of *P. putida* wild type (blue) and PSAG-9 (yellow) in MOPS with different P sources. **B** Growth of *P. putida* wild type (blue) and PSAG-9 (yellow) in LB with and without Pt. Growth was monitored by measuring OD_600_ in 500 mL flasks. Error bars represent the standard deviation between biological triplicates in each condition

The growth of PSAG-9 was examined in flasks (hence the density difference between the experiments depicted in Figs. 2 and 3) to verify the auxotrophic nature of the strain in both minimal and rich media using the wild type KT2440 as a control. As expected, PSAG-9 was not capable of growing in MOPS-Pi or in LB lacking Pt (Fig. 3). To further investigate the growth of PSAG-9 in a Pt environment compared with the wild type in Pi, the growth rate of the two strains was calculated in different media. PSAG-9 in MOPS-Pt and LB-Pt was 0.51 ± 0.1 and 0.44 ± 0.02 (n = 3, mean ± sd) which was about 100% of the growth rate of the wild type on MOPS-Pi and 57% of that on LB, respectively. Regarding final growth, at 32 h PSAG-9 had reached in MOPS-Pt an 87.5% of the final OD_600_ of the wild type on MOPS-Pi. In LB-Pt however, the final OD_600_ of PSAG-9 was only 67.1% of that reached by the wild type strain in LB. These results revealed that PSAG-9 is not able to make use of Pi in either inorganic or organic forms but can consume Pt in a similarly effective manner to the wild type when making use of Pi, this being more evident in minimal media.

#### Phenotypic characterization of the robustness of PSAG-9 as a chassis strain

**Fig. 4:**
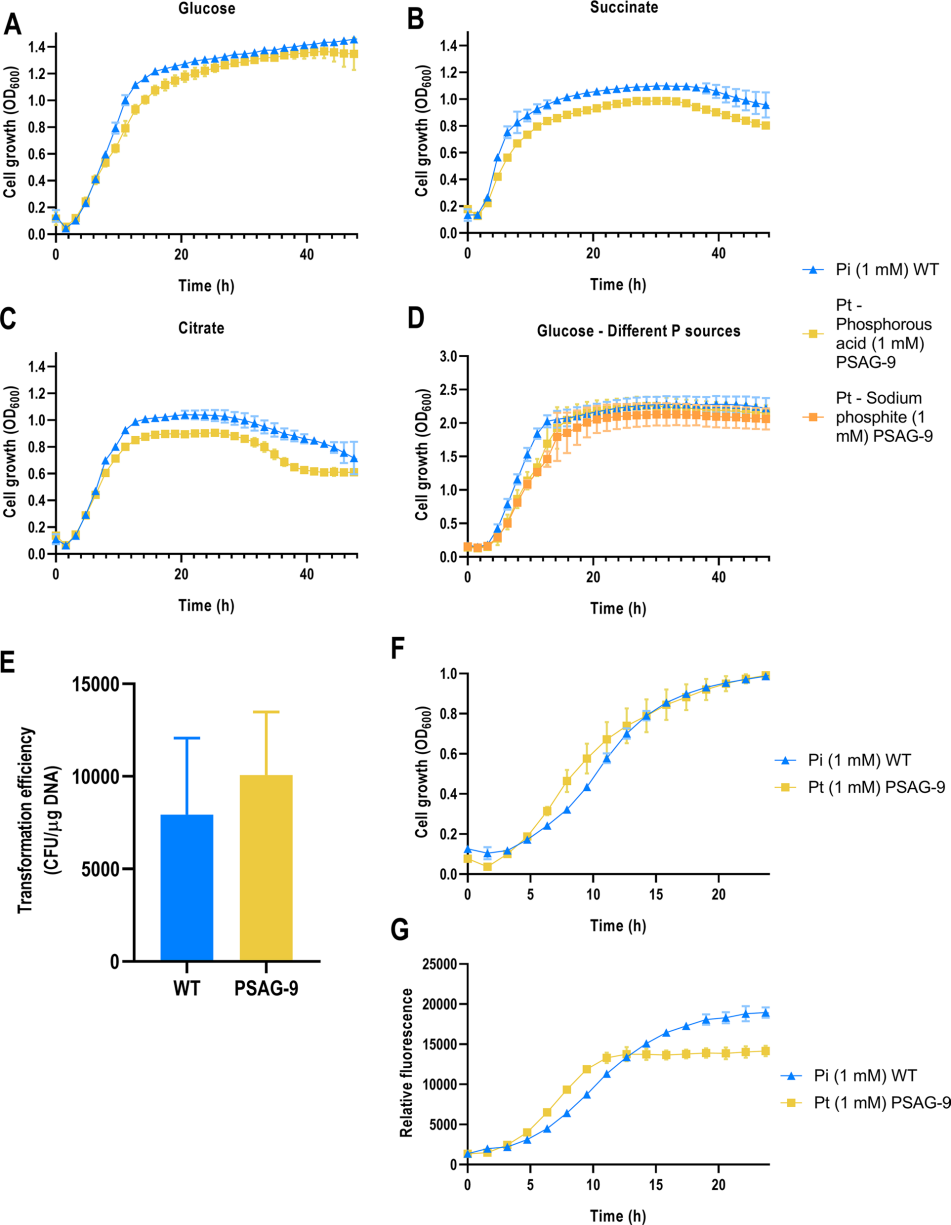
Phenotypic characterization of PSAG-9. Growth in MOPS supplemented with 50 mM glucose (**A**), 50 mM succinate (**B**), and 50 mM citrate (**C**). **D** Growth in MOPS supplemented with 50 mM glucose to test different Pt sources. As P sources, Pi (triangle) was used to grow *P. putida* wild type, and Pt (square) was used to grow *P. putida* PSAG-9, employing for this purpose either phosphorous acid (yellow) or sodium phosphite (orange) as Pt source. **E** Comparison between the transformation efficiency in *P. putida* KT2440 and *P. putida* PSAG-9. Transformation efficiency was calculated using the expression vector pSEVAb23 pAND105 sfGFP. CFU count was performed in plates containing MOPS-glucose agar containing either 1 mM Pi (wild type) or 1 mM Pt (PSAG-9) 48 h after electrotransformation of the strains. **F** Growth of strains harboring the expression vector pSEVAb23 pAND105 sfGFP, in MOPS supplemented with 50 mM glucose and corresponding P sources. **G** Relative fluorescence of strains harboring the expression vector pSEVAb23 pAND105 sfGFP. Data represent normalized fluorescence levels, expressed as a ratio with the OD_600_ over 24 h. Growth and fluorescence were monitored using a Synergy Mx Multi-Mode Microplate Reader or an Epoch 2 Microplate Spectrophotometer (BioTek Instruments, Inc., VT, U.S.). Error bars represent the standard deviation among biological duplicates and technical triplicates for each condition

To test the robustness of the strain, we evaluated the growth of PSAG-9 in minimal MOPS media with other C sources that impose a different metabolic regime, namely the gluconeogenic succinate and citrate. As it had been observed in MOPS-glucose (Figs. 3A, 4A), PSAG-9 showed the same lag phase than the wild type with slightly lower growth rates and final OD_600_, both in MOPS-succinate (Fig. 4B) and MOPS-citrate (Fig. 4C). In the same vein, we tested in MOPS-glucose an alternative source of Pt for PSAG-9, sodium phosphite, which confirmed that the results we were obtaining with phosphorous acid were reproducible with other Pt origins (Fig. 4D). To further validate the use of this strain as an interesting chassis for synthetic biology applications, we evaluated the transformation efficiency, the growth when harbouring plasmids and the fluorescence levels produced by the reporter GFP of PSAG-9 compared to the wild type. These three assays were performed in MOPS-glucose media supplemented with the corresponding P sources and antibiotics. In the first place, transformation efficiency of the expression vector pSEVAb23 pAND105 sfGFP into PSAG-9 showed no significant differences with that into the wild type control (Fig. 4E). Second, both mutant and wild type strains grew in a similar manner when carrying the aforementioned expression vector, with no major discrepancies in lag phase and final OD_600_, and only a slightly higher growth rate of PSAG-9 (Fig. 4F). Lastly, we proved that PSAG-9 is able to express reporter genes like *gfp*, even though the final relative fluorescence levels observed in this case for the mutant strain were 75% of those of the control (Fig. 4G).

### The auxotrophic PSAG-9 strain strictly uses Pt as the only P source

**Fig. 5:**
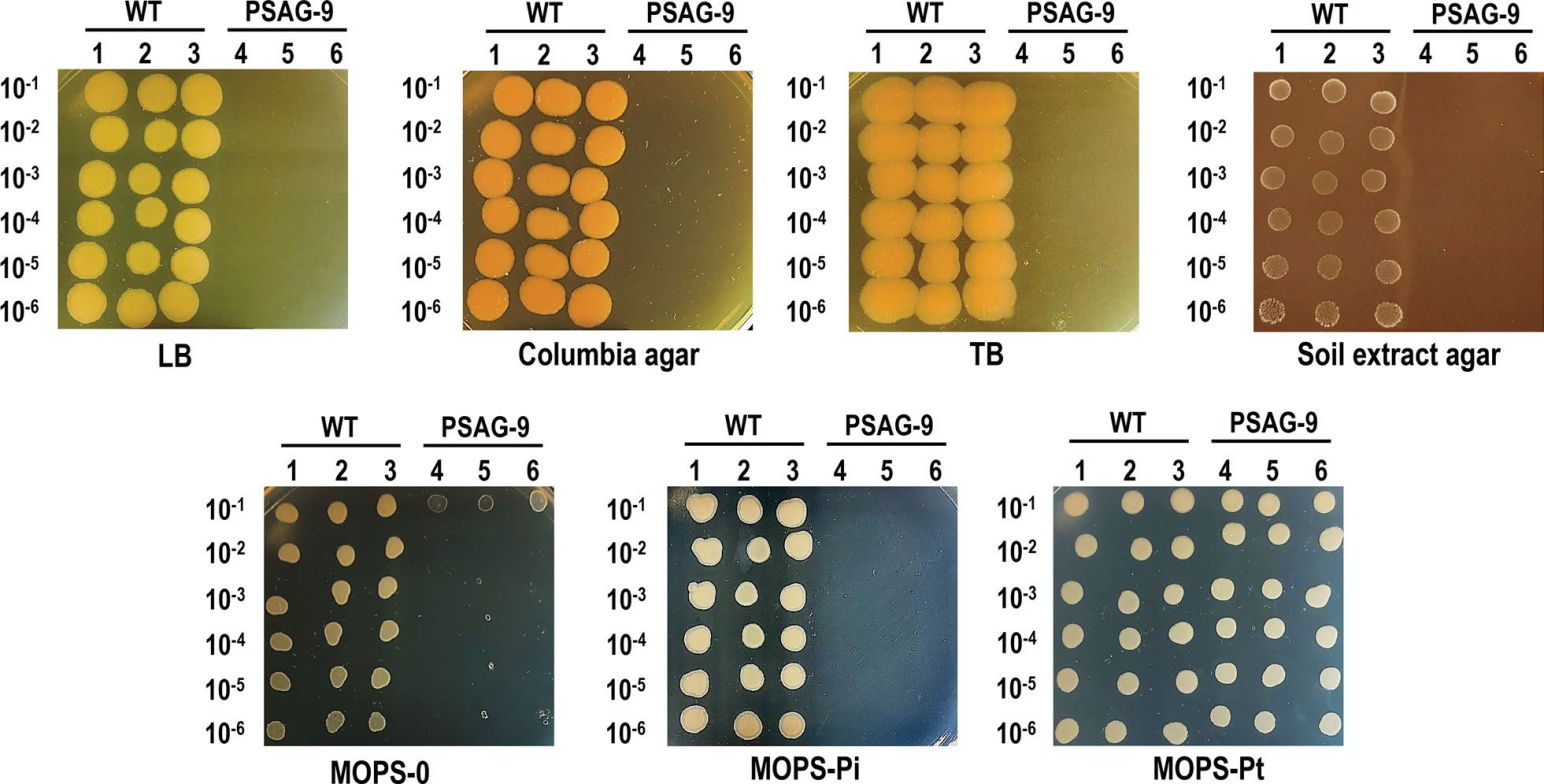
PSAG-9 could not use other various P sources except Pt. A spot assay was performed on seven different types of agar-containing media: LB, Columbia, TB, Soil extract, MOPS-0 (P-free MOPS-glucose), MOPS-glucose-Pi, and MOPS-glucose-Pt. Three biological replicates were plated in different dilutions for the wild type (1, 2 and 3) and PSAG-9 (4, 5 and 6) respectively. For the spots, dilutions from an overnight culture ranging from 10^–1^ to 10^–6^ were used. Pictures were taken after 21 days of incubation at 30 °C

After producing a complete auxotrophic strain in liquid MOPS and LB media, a spot assay was carried out for a prolonged period of time to investigate if PSAG-9 was ultimately able to utilize other P sources present in different rich media (Fig. 5). Results showed that the P sources (supplied by the yeast extract, the different digests, maize starch, phosphate buffers, etc.) from different rich media typically used in our laboratory (LB, Columbia agar, and terrific broth (TB)) could not support the growth of PSAG-9 even after 21 days. In light of the native habitat of *P. putida*, Soil Extract Agar was also included in this experiment alongside the aforementioned rich and MOPS media. Soil Extract has a complex composition and typically contains plenty of nutrients required for the growth of soil bacterium [40, 41]. Nevertheless, PSAG-9 was also not able to grow on the Soil Extract Agar and only did so on MOPS-Pt. These results further demonstrated that PSAG-9 is incapable of using any organic or inorganic P source apart from Pt, corroborating that it was a fully Pt-dependent auxotrophic strain.

The wild type strain could grow on every media, including MOPS-Pt, despite its tested incapability to proliferate in liquid MOPS-Pt medium. This was also the case for the wild type *E. coli* MG1655 strain [23] and is most likely a consequence of internally accumulated Pi. Strikingly, PSAG-9 showed very limited growth on MOPS-0 at the 10^–1^ dilution. However, the results of our previous tests in liquid MOPS-0 and the general incapability of organisms to grow without P, rendered this phenomenon more an experimental artefact than actual viability of the mutant strain in absence of a P source.

### PSAG-9 presents a very low escape frequency on soil media

Subsequently, we studied the escape frequency of PSAG-9 in Soil Extract Agar under non-permissive conditions (without Pt). Once again, we selected Soil Extract Agar as the medium of our experiment to determine the biosafety credentials of our strain in the most contextual conditions [19]. To do so, we started with a cell culture of 1 L MOPS-Pt containing ~ 3.6 × 10^13^ cells, which was plated onto both Soil Extract Agar-Pt and Soil Extract Agar-Pi plates. No escapers were observed during the first week of incubation. However, CFU were counted after 21 days resulting in an escape frequency of 2.05 × 10^–10^ ± 2.76 × 10^–10^ (n = 3, mean ± sd). Escapers were verified as *P. putida* PSAG-9 and were henceforth able to grow in both permissive and non-permissive MOPS media.

### *P. putida* PSAG-9 offers other industrially beneficial traits beyond biosafety

Given the potential of *P. putida* as an industrial chassis, we used PSAG-9 to test other beneficial traits that could be attractive for industrial applications besides biosafety.

#### Pt auxotrophy allows cultivation under non-sterile conditions

**Fig. 6:**
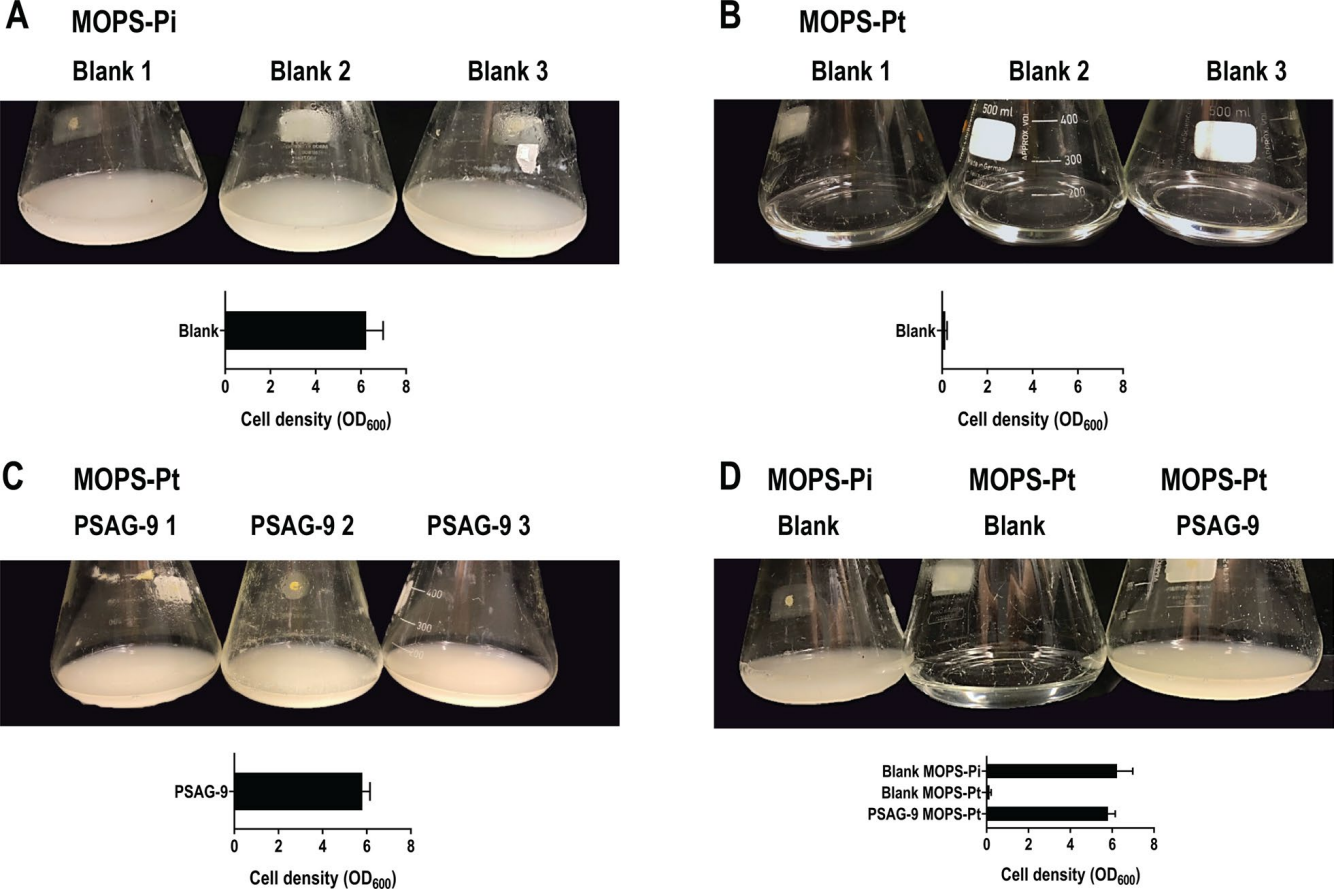
Pt as sole P source effectively inhibited the growth of other biological contaminants. PSAG-9 clones were cultured under non-sterile conditions in 250 mL flasks. Blank cultures of MOPS-Pi and MOPS-Pt were included as controls and each group was incubated in triplicates. Pictures were taken after 5 days of incubation at 30 °C and 200 rpm. **A** The blanks with only MOPS-Pi medium; **B** the blanks with only MOPS-Pt medium; **C** the growth of PSAG-9 at 5 days post-inoculation in MOPS-Pt; and, **D** comparison of the three groups

Strains that offer the possibility to operate under non-sterile conditions have become one of the most commercially appealing options when it comes to industrial production [42, 43]. It is already known that Pt is barely available in the environment and therefore can hardly be used by other organisms [24]. As a consequence, the Pt auxotrophy was expected to render PSAG-9 able to perform non-sterile fermentation with a reduced risk of biological contamination. To verify this, a non-sterility test was performed by using clean but non-sterilized flasks. While the employed media was initially sterilized, all subsequent steps were done under non-sterile conditions including inoculation. We observed that the blank of MOPS-Pt remained clear after 5 days of incubation (Fig. 6B) while the blank of MOPS-Pi showed contamination since the second day of the experiment (Fig. 6A). Meanwhile, the turbidity of the flasks inoculated with PSAG-9 was significantly increased after 5 days of incubation (Fig. 6C). The results indicated that the media with Pt as the only P source was effectively not suitable for the growth of other microorganisms.

To further investigate the bacterial growth from the two turbid groups (initially, blank MOPS-Pi and MOPS-Pt inoculated with PSAG-9), samples were diluted and spread onto plates and microbial diversity was distinguished by microbial morphology. On the one hand, the colonies that grew from the blank MOPS-Pi flask presented bigger and more yellowish morphologies when compared to PSAG-9 (Additional file 1: Figure S3, left). Various colonies were chosen arbitrarily and subjected to specific PCR amplification confirming that none of them showed the PSAG-9 genotype. On the other hand, the morphology of the colonies derived from MOPS-Pt-PSAG-9 was more uniform and resembled the typical characteristics of the PSAG-9 (Additional file 1: Figure S3, right) indicating that the group MOPS-Pt-PSAG-9 only contained the strain that was initially inoculated. This was subsequently verified by PCR amplifying 8 colonies, which positively showed the expected genotype.

#### The phosphite assimilation pathway provides reducing power

**Fig. 7:**
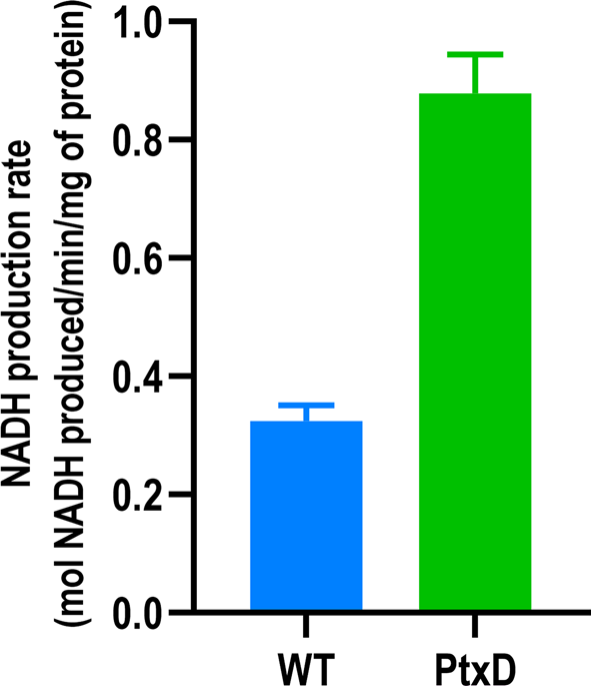
NADH production rate in wild type (blue) and PtxD-containing (green) cell extracts. Wells were seeded with 0.3–0.45 mg/mL of total protein from clarified cell extracts. Values were normalised for total protein concentration in each extract. OD_340_ was measured for 90 min in 30-s intervals and the depicted bars represent the increasing NADH concentration rate throughout that period. Error bars represent the standard deviation between biological triplicates and technical duplicates in each condition

The NAD^+^-dependent Pt dehydrogenase PtxD produces NADH during the conversion of Pt to Pi [44]. Given the high redox potential of the NADH/NAD^+^ couple and its value as metabolic currency to carry out NADH-dependent metabolic processes, we investigated whether *P. putida* PSAG or its derivates could generate additional NADH when growing on Pt. This production of NADH on a source of Pt would also give an indication that PtxD functionally converts Pt to Pi, and detecting NADH production would provide a suitable measure of the activity of the enzyme. To examine this, cell extracts were prepared from both *P. putida* KT2440 and *P. putida* PSAG cell cultures to follow their NADH production over time (Additional file 1: Figure S4). The rate of increasing NADH concentration per mg/mL of protein was calculated as 0.87 mol/min/mg of protein for the PtxD-containing cultures. Interestingly, extracts of cells deficient in PtxD also showed a NADH increase of around 0.326 mol/min/mg of protein of protein, suggesting that the wild type could have a native Pt dehydrogenase activity, although significantly less efficient than PtxD (Fig. 7).

## Discussion

### Assimilation of P in* P. putida*

We successfully engineered a phosphite synthetic auxotrophy in *P. putida* KT2440. Our research showed that the auxotrophic strain PSAG-9, which contains in its genome the *ptxD* and *htxBCDE* genes, can effectively uptake Pt as a P source for supporting growth (Fig. 2A). Furthermore, the deletion of all native Pi transporters including the inorganic Pi transporters, Pn transporter, organic Pi transporters, and the PhoBR regulatory system suppressed the ability of the strain to utilize Pi (Fig. 3). Ultimately, no growth of PSAG-9 was observed in any medium lacking Pt (Figs. 3, 5). Prior to this research, references to the native Pi transport system of *P. putida* KT2440 were scarce [45, 46], and most annotations of transporters were based on their homology with their *E. coli*’s counterparts. During this study, we found that deletions in what we identified by homology as PitA (*PP_4103*) did not dramatically affect the growth of *P. putida* in Pi (Additional file 1: Figure S2A). In *Mycobacterium smegmatis*, deletions of PitA similarly did not alter phosphate uptake and cell growth, even though PitA is the only low-affinity Pi transporter annotated in this organism [47]. However, the deletion of PitA in *M. smegmatis* was found to cause the upregulation of PstSCAB. This further supports the notion that Pi assimilation relies on the redundancy of regulatable transporters, and that PstSCAB alone is likely sufficient to support growth. This aligns with our results with *P. putida*, which suggested that the strain could still grow efficiently after the deletion of both PitA and PitB (Fig. 2B). Furthermore, only the PstB component of the second PstSCAB complex was annotated as *pstB-II* at the beginning of our research. First, we hypothesized the possibility of PstB-II being a single isoenzyme of PstB. However, we ultimately concluded, based on the homology of the neighbouring genes, that the operon *PP_5326-PP_5329* could be a completely separate PstSCAB complex. It should be noted that only when both *pstSCAB* and *pstSCAB-II* operons were completely deleted the strain was unable to grow on Pi in minimal medium, confirming our latter hypothesis.

In the environment, many species are able to fulfil their P requirements through import of organic Pi-esters, including phosphonates. Around 10% of the dissolved P in the ocean is in a phosphonate form, and some marine life has adapted to assimilate it [48]. Phosphonates are also known to be utilised by soil bacteria. Specifically, *P. putida* has been demonstrated to utilise the phosphonate 2-aminoethylphosphonic acid as its sole source of carbon, nitrogen and P, further demonstrating the robustness of this organism’s metabolism [49]. Interestingly, the presence of Pt specifically inhibited the activity of the enzymes required to convert the phosphonate into Pi. Regardless, we still removed *P. putida*’s ability to transport phosphonates by deleting the *phnCEptxBC* genes to ensure that the organism could not grow on any other environmental form of P other than Pt.

Regarding the exact function of the deleted *PP_3818*, little is known beyond its annotation as a periplasmic phosphate transport system substrate-binding protein. While it contains an outer membrane protein A (OmpA) domain, much like the phosphate scavenging protein 3 (Psp3 found in comparative genomic analysis of *Pseudomonas* isolates [50], there is no evidence of the product of *PP_3818* having this scavenger function. Nevertheless, exoproteins Psp, secreted under Pi-limited conditions to scavenge extracellular Pi before being transported back into the cells through the PstSCAB transporters [51], which had been previously knocked-out of our strain’s genome. On a different note, homologs of the glucose-3-phosphate transporter genes are widely found in many living organisms and their function had been previously characterized in *E. coli* [52, 53], revealing that on top of glycerol-3-phosphate, this transporter may participate as well in Pi uptake [54]. In contrast, the existing literature on the Na^+^/Pi symporter YjbB is limited but the few studies on *E. coli*’s YjbB suggest that it might also be involved in the transport of Pi and the regulation of the pho regulatory genes [37]. The PHO Pi starvation regulatory system has recently been studied in depth in *P. putida* [55]. The mechanism involves the response regulator PhoB and the histidine kinase PhoR, which were known in *P. putida* KT2440 for regulating the expression of a series of genes related to Pi transport or assimilation under inorganic Pi starvation [38].

The main effect of Pho induction is the upregulation of *pstSCAB* to compensate for the low internal Pi concentration, which may explain why the deletion of *pitB* and *pitA*, and the likely initial condition of Pi starvation that the deletions caused, resulted in an increased ability to grow on Pi (Fig. 2B). Similarly, the lack of growth defect in Pi upon *pstSCAB* deletion (Additional file 1: Figure S2B) may have been a result of the upregulation of the second *pstSCAB-II* variant as a way of compensation. As a result of this Pi concentration-dependant feedback system, it would be difficult to calculate the relative effect that each of the sequential deletions actually has on the wild type KT2440. Although a major goal of this project was to eliminate growth in Pi, which required the removal of all Pi transporters, it would be useful to measure the effect of each transporter deletion on the resulting upregulation and expression of the other transporters to verify our initial assumption, which could be done through a comparative RNASeq analysis to establish the relative abundance of mRNA for the transporters in each knock-out configuration [56]. This, however, remains out of the scope of this work, given that our focus was on the establishment of the biocontainment strategy, which we successfully achieved by targeting the aforementioned elements and transporters.

### Characteristics of PSAG-9 and its biosafety credentials

Notably, as it can be seen in Figs. 3 and 5, the wild type *P. putida* could grow slightly in both liquid and solid media without P source or Pt as the only P source. In this regard, previous studies have found that *P. putida* has the ability to survive under phosphate starvation [57]. There are probably two reasons to explain this. First, the active aforementioned PHO starvation regulatory system, which results in the strong Pi storage capacity of the strain accumulating it in the form of polyP. There is evidence that these polyPs play an important role in the cells under stress conditions [32]. Second, the phosphate-deficient environment exerts strong selection pressure on *P. putida*, which can lead to the development of mutants that have a growth advantage in the environment and are able to grow with nutrients from dead cells [57]. However, this was not the case with PSAG-9, suggesting that the phosphite auxotrophy strategy weakens the tolerance of PSAG-9 to P starvation to a great extent, ensuring that the viability of PSAG-9 will be rapidly reduced even when PSAG-9 is released to the open environment. On the other hand, the growth assay (Fig. 3) reflected that although the growth rate of PSAG-9 in the presence of Pt was similar to the wild type in Pi during the growth phase, its final cell density was lower than that of KT2440. This difference was even more pronounced for the strains growing in rich LB medium, indicating that the engineered Pt auxotrophy has a certain effect on bacterial fitness. The stationary phase usually occurs when nutrients are not enough to support cell division [58]. As mentioned above, the wild type is more tolerant to P starvation than PSAG-9, therefore, the former is always able to survive better than PSAG-9 when there is no supplementation of any P source. Considering that LB is a rich medium, we speculated that LB contains a higher concentration of P source to support the growth and proliferation of the wild type than MOPS medium does when supplied only with an inorganic P source. Since the P source of PSAG-9 is still Pt, the difference between the two strains in LB at the stationary stage should be expected to be more significant. In addition, we hypothesized that the presence of heterogenous P sources and nutrients in the LB-Pt medium might result in the activation of genes (i.e., P scavengers and probably other P unrelated genes too) that ultimately are not able to properly carry out their function due to the deletion of the pho regulon. The activation of all these dysregulated genes could cause a burden in the cells, resulting in the observed reduced fitness. Nonetheless, this phenomenon needs to be further investigated to draw clearer conclusions.

Furthermore, our study showed that PSAG-9 cannot survive under any non-permissible conditions (Figs. 3, 5). In the absence of Pt, the probability of occurrence of escape mutants surpassed the order of 10^–10^. For the practical application of *P. putida* as a synthetic biology chassis, the lower the escape frequency, the higher the guarantee for biosafety. The fact that phosphite auxotrophy applied in *P. putida* yielded higher escape frequency as compared to that in *E. coli* [23] may reflect that *P. putida* may be better coping with P starvation [57] or that its rich metabolism and intrinsic redundancy may be a disadvantage in the context of this strategy. Nonetheless, we have to take into account that the escape frequency here reported was obtained on a different media than the employed for other microorganisms which hinders any valid comparison. Previous studies used minimal media to report this metric, whereas we have utilized Soil Extract Agar, given some of the envisioned applications of our phosphite auxotroph [19].

Given that the observed escape mutants appeared after a considerably long period, we reason that strain PSAG-9 does not bear any other phosphate-specific transport mechanism. As such, its regained ability to grow on P sources is most likely resulting from a series of acquired evolutionary mutations. Since our modifications have not affected the core Pi metabolism of the mutant but only its transport, we believe that these mutations may have altered the structural configuration of other native transporters specific to molecules such as sulphate (similar in size to phosphate), or promiscuous transporters with the ability to cotransport different molecules [46]. As a result, these mutations could either unlock new transport abilities or improve the specificity of a low affinity transporter to incorporate Pi. *P. putida* carries a plethora of transporters (mostly of unknown substrate specificity), including 18 ferric and ferric-related siderophore-receptors, two sulfonate ABC transport systems, various transporters for sulphate, two for nitrate, plus several other transport systems for common metals [5, 59]. The high abundance of multiple transport mechanisms, especially for iron and phosphate is consistent with *P. putida*’s potential to overcome nutrition limitations in diluted environments.

### PSAG-9 is biocontained, can be cultivated under non-sterile conditions and can provide additional reducing power

As a biocontainment strategy, Pt synthetic auxotrophy provides many advantages. Pt is cheap, non-toxic, simple to produce and recyclable, and as such would make an acceptable supplement for large quantities of culture media [60]. Pt is a stable compound and is not likely to be depleted by the majority of organisms that might share an environment with the auxotrophic *P. putida*. In addition, its centrality to the maintenance of the auxotrophic strain, in being required for nucleic acid and ATP synthesis, suggests that microbes are less likely to be able to circumvent this biocontainment strategy, as compared to other that encode, for example, toxin: antitoxin pairs. The PSAG-9 strain constructed in this project not only guarantees its biosafety as a chassis, but it does so more cheaply. Indeed, as compared to the vitamins and amino acids commonly used in auxotrophy strategies, the costs of feeding Pt are extremely low [23]. In this research, we demonstrated that PSAG-9 works as a valid and robust synthetic biology chassis, able to operate with different C and Pt sources (Fig. 4). Its transformation efficiency and fitness when harbouring plasmids do not differ from those of the wild type, although we observed affected expression levels when monitoring GFP-emitted fluorescence (Fig. 4). This latter phenomenon should be further investigated in future research by means of alternative reporters and protein and metabolite production experiments.

Furthermore, due to cultivation characteristics where a typically not metabolizable nutrient as Pt is supplemented as sole P source, the strategy implemented in PSAG-9 has the effect of controlling contamination from other undesired bacteria (Fig. 6). This feature provides the potential for large-scale non-sterilized fermentation, saving the cost and time of sterilization of equipment and media in industrial production.

Moreover, as a by-product of the Pt conversion to Pi by the dehydrogenase PtxD, NAD^+^ is reduced to NADH [44]. The reducing power of NADH makes it a valuable metabolic currency with uses in many catabolic processes [61]. *P. putida* already has an intrinsic high rate of NADH regeneration [62]. High NADH availability is vital to the strategy of this bacterium in removing toxic compounds, as oxidation of the cofactor supplies the energy required to drive the organism’s efflux pumps [63]. In this study, we verified that PtxD-dependent conversion of Pt to Pi in *P. putida* could serve as a source of additional NADH to the already high pool of this cofactor (Fig. 7). However, we acknowledge that the amount of reduced cofactor obtained from 1 mM Pt is inconsequential when compared to the reducing power provided by glucose assimilation. Phosphite, nevertheless, is still considered as one of the most prominent electron donors for biocatalysis given its high solubility and low reduction potential [64]. Upon increasing demands of NADH and elevated Pt concentrations (20 mM), previous studies have demonstrated that the contribution of PtxD can result in significantly higher amounts of intracellular NADH levels [65]. Moreover, this would be a great benefit to *P. putida* strains encoding this auxotrophy that are designed to have applications in environmental bioremediation, as the Pt conversion provides additional energy to drive the catabolism of toxic compounds in the form of more available NADH.

Although PSAG-9 has numerous benefits and application potential, there is further research and optimization needed to overcome some of the strain’s limitations. First, only a single biocontainment strategy was applied to this strain. The study has shown that horizontal gene transfer might be a risk for this strategy because it can enable Pt-dependent strains to regain the ability to transport Pi if any Pi transporter is acquired horizontally [23]. Consequently, it would be convenient to combine this strategy with other safeguards for PSAG-9 to make up for the inherent shortcomings of the individual protection measures. For example, the Pt auxotrophy established in *P. putida* could be complemented with a methanol auxotrophy, meaning two entire auxotrophy systems would have to be circumvented to allow environmental escape [66]. More extreme changes, such as the establishment of sematic containment, could also be considered in future combinatorial biocontainment strategies for *P. putida*, in an effort to demonstrate biosafety to a level acceptable to regulators [67, 68]. Secondly, Pt is still widely used in agriculture as a fungicide [69]. If PSAG-9 is to be used for bioremediation caused by pesticides pollution, fields should be screened for whether they contained Pt or not. Since the strategy would only work optimally when Pt is present at the permissive condition areas, Pt removal from non-permissive sections could increase the workload of the overall cleaning procedure. Finally, research has suggested that the microbial community in soil and water is usually capable of rapidly absorbing and oxidizing Pt [23]. Whether the extensive use of Pt in the open environment supplied for the use of PSAG-9 will damage the balance of the original microbial community and cause the enrichment of the community remains to be verified.

### Orthogonality and concluding remarks

As a final remark, we would like to reflect on the orthogonality of genetic safeguards. While we may have ambitions towards having a repertoire of synthetic biology chassis that can be equipped on-demand with different standard modules and genetic safeguards, each of these features will still require accounting for their corresponding cellular and application contexts [19]. In this specific case, although it was fair to assume that the PSAG components described in the previous studies in *E. coli* and *S. elongatus* would function sufficiently in our chosen organism, it quickly became evident that bacterial species have distinct variations concerning their methods of Pi transport. In *Pseudomonas* alone, several species encode different combinations of Pi transporters [50]. Additionally, our initial search for transporters suggested that Pi transport in *P. putida* had not been detailed exhaustively, with many Pi-interacting proteins remaining unannotated and uncharacterised in the species. Similarly, the native Pi transporters of *E. coli* were disrupted by P1 transduction [23]. Our chosen method of gene deletion in *P. putida*, based on the integrative plasmid pGNW, required more technical steps and periods of overnight incubation to complete than P1 transduction, making the enactment of this biocontainment strategy in our chosen organism more time and resource intensive [70, 71]. As much as synthetic biology strives to simplify living systems, there is so much that we do not fully understand yet. The independence that an orthogonal element requires should, however, be possible only by fully comprehending the framework within which it operates.

In summary, by developing a biocontainment strategy based on synthetic auxotrophy for *P. putida*, this work attempted to enable its deployment as a synthetic biology chassis with potential uses in industry with enhanced, built-in biosafety features. Additionally, this may also provide a basis for its possible use in non-contained environmental applications.

## Methods

### Strains and growth conditions

All bacterial strains used in this study are listed in Additional file 1: Table S1. Culturing of bacterial strains was conducted in Lysogenic Broth. Strains carrying antibiotic selection markers were cultured with their respective antibiotics in the following concentrations: kanamycin 50 μg/mL, gentamicin 10 μg/mL and 15 μg/mL (for *E. coli* and *P. putida* respectively), apramycin 50 μg/mL and chloramphenicol 20 μg/mL. Morpholine propansesulfonic acid (MOPS) synthetic medium supplemented with 50 mM glucose was used as a minimal medium [72] for all phosphite synthetic auxotrophy experiments. Pt stock solutions were prepared dissolving phosphorous acid in distilled water followed by pH neutralization with NaOH, filtration and storage at − 20 °C. A pH neutralized and autoclaved K_2_HPO_4_ solution was used as a source of Pi [72]. MOPS-glucose media containing Pi and Pt are designated as MOPS-Pi and MOPS-Pt respectively. P-free MOPS-glucose medium is designated as MOPS-0. *E. coli* and *P. putida* strains were grown at optimal temperatures, 37 °C and 30 °C respectively.

### Plasmids

A list of all plasmids utilized in this study with their respective features is provided in Additional file 1: Table S2. Primers used for the amplification of genetic parts, genome editing verification and sequencing purposes (Additional file 1: Table S3) were synthesized by Integrated DNA Technologies (IDT) as salt-free without further purification, resuspended in milli-Q at 100 µM and long-term stored at − 20 °C. Q5® High-Fidelity DNA Polymerase (New England Biolabs) and Platinum SuperFi II DNA Polymerase (Thermo Fisher Scientific) were used for PCR amplification of genetic parts according to manufacturers’ instructions. The resulting PCR products were run on 1% (v/v) agarose gel to verify fragments size. PCR fragments were isolated from PCR product or from agarose gel using NucleoSpin® Gel and PCR Clean-up (BIOKÉ).

All plasmids were assembled using Golden Gate or the Golden Gate-based SevaBrick Assembly [73]; therefore, BsaI recognition sites were included in the primers to produce 4-nucleotide overhangs on the 5′- and 3′-ends of each PCR amplicon used for plasmid construction. Assembled plasmids were transformed into chemically competent *E. coli* DH5α using heat shock transformation [74]. Transformant *E. coli* colonies were subsequently selected and correct assembly of the constructs was screened via Phire Hot Start II DNA Polymerase (Thermo Fisher Scientific) colony PCR according to manufacturer’s instructions. Corresponding clones were inoculated in LB liquid cultures and plasmids were isolated using GeneJET Plasmid Miniprep Kit (Thermo Fisher Scientific) following manufacturer’s protocol. Plasmid insert sequences were ultimately confirmed with standard DNA sequencing (MACROGEN Europe).

Expression plasmids were transformed into *P. putida* via electroporation [75]. Cells were made electrocompetent by consecutive washing steps of 10, 2, and 1 mL of sucrose 300 mM. The washed cultures were finally resuspended in 200 μL of sucrose 300 mM. 100 μL of electrocompetent cells were transformed with 1 μL of recombineering oligo (100 μM). For assessment of transformation efficiency, CFU were counted in appropriate plates after 48 h and calculated per 1 μg of plasmid DNA.

### Engineering knock-out and knock-in* P. putida* mutants

Phosphite assimilation genes were introduced in the genome of *P. putida* KT2440 via site-specific recombination. First, *ptxD* from *Ralstonia sp.* 4506 under the control of the tac promoter [76] and *htxBCDE* from *P. stutzeri* WM88 (Additional file 1: Table S4) under the control of the lac promoter were amplified from plasmids pSTV28 with primers C3 and C4, and C5 and C6, respectively, and cloned together in pSEVAb84 ptxD + htxBCDE, using the pSEVAb84 backbone amplified with C1 and C2 primers. Either *ptxD* only, by using primers C11 and C12, or the whole cassette, using primers C11 and C13, were amplified for construction of pSEVAb22 cre + ptxD and pSEVAb22 cre + ptxD htxBCDE, using the in-house amplicon pSEVAb22 cre which was previously amplified with C9 and C10 and works as a standard backbone for *cre lox* integration in a *lox*71-*lox*66/2 m landing pad located in the *att*Tn7 locus of the *P. putida* genome. Transformation of pSEVAb22 cre plasmids was performed via electroporation in competent *P. putida* KT2440 [75] cells containing the mentioned landing pad. Site-specific recombination and subsequent cassette integration was confirmed with V1 and V2 primers and resulted in the creation of the *P. putida* PSAG strain.

*P. putida* PSAG-derived knock-out and knock-in strains were generated as previously described using tri-parental conjugation for chromosomal integration of suicide pGNW vectors followed by the action of meganuclease I-SceI, which was located in a pQURE6.H plasmid [71, 77]. Construction of pGNW plasmids for knock-outs was based on the cloning of an upstream and a downstream homologous regions of 500 bp required for homologous recombination into the pGNW backbone amplified with P1 and P2 primers. Individual *P. putida* clones were tested for wild type or mutant genotype via Phire Hot Start II DNA Polymerase (Thermo Fisher Scientific) colony PCR according to manufacturer’s instructions with corresponding verification primers.

Following the aforementioned knock-out protocol, all endogenous phosphate transport encoding genes were sequentially knocked-out of the *P. putida* PSAG genome including those that encode: (a) the low affinity inorganic phosphate transporters PitA and PitB (*PP_4103* and *PP_1373*); (b) the high affinity inorganic phosphate transporters PstSCAB and PstSCAB-II (*PP_2656*-*PP_2659* and *PP_5326*-*PP_5329*); (c) the phosphate and phosphonate transporter complex PhnCE-PtxBC (*PP_0824*-*PP_0827*); (d) the periplasmic phosphate-binding protein encoded by *PP_3818*; (e) the glycerol-phosphate transporter encoded by *PP_2260*-*PP_2063*; (f) the Na + /phosphate symporter YjbB (*PP_0145*); and (g) the two-component regulatory system PhoBR (*PP_5320*-*PP_5321*).

### Growth and fitness assays

To determine the growth and fitness of the different *P. putida* PSAG strains in different P sources, assays were performed on a smaller and a larger scale. Precultures of *P. putida* KT2440 and PSAG strains were prepared 18–24 h prior the harvesting which was done by centrifugation at 4700 g for 5 min at room temperature. Pellets were washed twice with an equal volume of MOPS-0, and finally resuspended in MOPS-0 to a final volume of 5 mL. The growth and fitness of the samples were monitored by measuring OD_600_. For the small-scale experiments, monitoring was performed for 24–72 h at 30 °C under gentle agitation in an ELx808 Absorbance Microplate Reader, a Synergy Mx Multi-Mode Microplate Reader, or an Epoch 2 Microplate Spectrophotometer (BioTek Instruments, Inc., VT, U.S.). Cultures were seeded in biological duplicates and technical triplicates to an initial OD_600_ of 0.3 in either MOPS-0, MOPS-Pi, MOPS-Pt, LB, or LB-Pt media, to a final volume of 200 μL per well in a 96-well plate. Large scale experiments were performed in 500 mL flasks. Each sample was inoculated into MOPS-0, MOPS-Pi, and MOPS-Pt to an initial OD_600_ of 0.2 in a final volume of 50 mL. Cultures were incubated at 200 rpm and 30 °C for 24 h. Growth was monitored by measuring OD_600_ every 1 to 1.5 h. The growth rate was calculated as a function of the variation in the number of cells during the exponential phase period.

To test the robustness of the final strain PSAG-9, growth was measured with other carbon sources, namely 50 mM succinate or 50 mM citrate. In addition, growth was also assessed with the additional Pt source sodium phosphite at 1 mM.

### Assessment of different P source viability

For assaying whether the *P. putida* PSAG strain strictly uses Pt as the only P source, the growth of the strain on different types of media containing a variety of P sources was examined. Three biological replicates of *P. putida* PSAG-9 and *P. putida* KT2440 were grown overnight. Precultures were pelleted down and washed with an equal volume of MOPS-0. Cells were resuspended in 5 mL MOPS-0 and bacterial resuspensions were seeded into a 96-well plate to a final volume of 200 μL with an equal OD_600_. 2 μL of corresponding serial dilutions were spotted onto different agar plates including Lysogenic Broth (10 g/L NaCl, 10 g/L tryptone, 5 g/L yeast extract and 15 g/L bacteriological agar), Columbia medium (10 g/L pancreatic digest of casein, 5 g/L meat peptic digest,3 g/L heart pancreatic digest, 5 g/L yeast extract, 1 g/L maize starch, 5 g/L sodium chloride, 12 g/L bacteriological agar), Terrific Broth (12 g/L tryptone, 24 g/L yeast extract, 0.4% (v/v) glycerol, 10% (v/v) phosphate buffer (23.12 g/L KH_2_PO_4_ and 125.4 g/L K_2_HPO_4_) and 15 g/L bacteriological agar), Soil Extract Agar (50 mM glucose, 1 g/L yeast extract, 10% soil extract, 15 g/L bacteriological agar [78]), MOPS-Pi, MOPS-Pt, and MOPS-0 [72]. Soil extract was prepared as follows: 250 g of wet soil collected at Wageningen Campus were resuspended in 1 L of distilled water, homogenised, autoclaved, filtered through a 0.2 µm-pore size filter and pH-adjusted to 7.0 [78]. The plates were incubated at 30 °C and growth of the spots was monitored for 21 days.

### Escape frequency assay

To examine the reliability of the Pt-dependent containment strategy on *P. putida*, escape frequency of auxotrophic strains was assessed in permissive and non-permissive soil conditions. Three biological replicates of the auxotrophic *P. putida* PSAG-9 strain were incubated at 30 °C overnight. Precultures were then used for inoculation of 1 L cultures in 5 L-flask of MOPS-Pt to an initial OD_600_ of 0.2. Next day, grown cultures were centrifuged at 4700 g for 10 min to collect the pellet. Cells were washed twice with an equal volume of MOPS-0 and resuspended in 1 mL MOPS-0 for subsequent preparation of serial dilutions down to 10^–15^. 50 μL from relevant dilutions was plated on Soil Extract Agar-1 mM Pi and Soil Extract Agar-1 mM Pt. Plates were grown for 21 days before colonies were counted. Escape frequency was calculated as the ratio between the number of colonies that escaped and could grow in Soil Extract Agar and the total number of colonies that grew in Soil Extract Agar-Pt [79].

### Non-sterile fermentation ability test

For the non-sterile fermentation test, cultures of 10 mL of *P. putida* PSAG-9 were grown in triplicates to an OD_600_ of 5 and were used to inoculate MOPS-Pt in 0.5 L flasks to a final volume of 50 mL with a starting OD_600_ of 0.1. Flasks of 0.5 L containing 50 mL of MOPS-Pt and MOPS-Pi were used in triplicates as blank during the experiment. The flasks and medium utilized in this experiment were not sterilized and the inoculation was performed under non-sterile conditions. Flasks were incubated at 30 °C and 200 rpm for 5 days. Samples of the cultures were collected every 24 h for plating and visual analysis of the grown microbial community.

### *NAD*^+^*/NADH conversion assay*

*P. putida* PSAG was grown to mid-exponential phase, alongside *P. putida* KT2440. Cells were harvested by centrifugation at 4700*g* for 10 min at 4 °C and resuspended in cold PBS buffer containing 10 mM 2-mercaptoethanol. The washed cells were centrifuged again under the same conditions, resuspended in 750 μL of the same PBS buffer, and transferred to bead-beater tubes containing 0.1 mm silica beads. Cells were subsequently lysed by pulsing with a cell disruptor for 30 s, and then cooled for 5 min on ice before being pulsed for a second time. The crude lysates were clarified by centrifugation at 7500*g* for 30 min at 4 °C before being transferred to a fresh 1.5 mL tube. The protein concentrations of the cell extracts were calculated by a 30–45-min incubation with Bradford reagent and comparing absorbance at OD_595_ with a BSA standard curve using a Synergy™ Mx microplate reader. To measure NAD^+^/ NADH conversion rates, a buffer solution containing 50 mM K_2_HPO_4_, 5 mM MgSO_4_ and 0.5 mM NAD^+^ were mixed with 40 μL of cell lysate solution from the wild type and ptxD containing cultures in a 96-well plate in biological triplicates and technical duplicates. 0.5 mM Pt was then added to the samples to a final volume of 200 μL, with water otherwise used as a negative control. Immediately after mixing, the plate was monitored for NADH production by measuring absorbance at 340 nm over 90 min. The OD_340_ values were then converted into NADH concentration by comparison with a prepared NADH standard curve and normalised for protein concentration.

### Fluorescence assay

*P. putida* KT2440 wild type and *P. putida* PSAG-9, both harbouring pSEVAb23 pAND105 sfGFP, were grown at 30 °C and 200 rpm, overnight in 10 mL MOPS-Pi and MOPS-Pt media, respectively, supplemented with kanamycin. Overnight cells were harvested at 4700 g for 10 min and washed twice with MOPS-0. Cells were resuspended to an OD_600_ of 0.3 and grown aerobically at 30 °C in fresh MOPS-Pi (wild type) and MOPS-Pt (PSAG-9) supplemented with 50 mM of glucose and kanamycin on 96-well black wall and transparent round-bottom plate in a total volume of 200 μL per well. Optical density (OD_600_) and green fluorescence (excitation 467 nm, emission 508 nm) readings were monitored in a BioTek Synergy Mx Multi-Mode Microplate Reader over 24 h (BioTek Instruments, Inc., VT, U.S.). Fluorescence values were normalized to OD_600_ values. Biological and technical triplicates were included.

## Supplementary Information

**Additional file 1.** Supplementary figures and tables.

## Data Availability

Not applicable.
