# Supplementary material for: Phosphite synthetic auxotrophy as an effective biocontainment strategy for the industrial chassis Pseudomonas putida
Source: Microb Cell Fact. 2022 Aug 8;21:156. doi: 10.1186/s12934-022-01883-5 (PMC9358898; doi:10.1186/s12934-022-01883-5)
Supplement: Supplementary file 1 — Additional file 1. Supplementary figures and tables. [file 12934_2022_1883_MOESM1_ESM.docx]

**Supplementary Information**

**Phosphite synthetic auxotrophy as an effective biocontainment strategy for the industrial chassis *Pseudomonas putida***

by

Enrique Asin-Garcia^1^ (https://orcid.org/0000-0001-5568-345X), Christos Batianis^1^ (https://orcid.org/0000-0002-2248-4523), Yunsong Li^1^, James D. Fawcett^1,^ ^[[1]](#footnote-1)^ (https://orcid.org/0000-0002-1797-6670), Ivar de Jong^1,^ ^[[2]](#footnote-2)^ (https://orcid.org/0000-0003-3239-9258), Vitor A. P. Martins dos Santos^1, 2, 3,^ * (https://orcid.org/0000-0002-2352-9017)

^1^ Laboratory of Systems and Synthetic Biology, Wageningen University & Research, Wageningen, 6708 WE, The Netherlands

^2^ LifeGlimmer GmbH, Berlin, 12163, Germany

^3^ Bioprocess Engineering Group, Wageningen University & Research, Wageningen, 6700 AA, The Netherlands

* To whom correspondence should be addressed. Tel: +31317482865; Email: [vitor.martinsdossantos@wur.nl](mailto:vitor.martinsdossantos@wur.nl)

**Supplementary Figure S1. The native Pi transporters of *P. putida* KT2440 have a Pt transport activity**

**
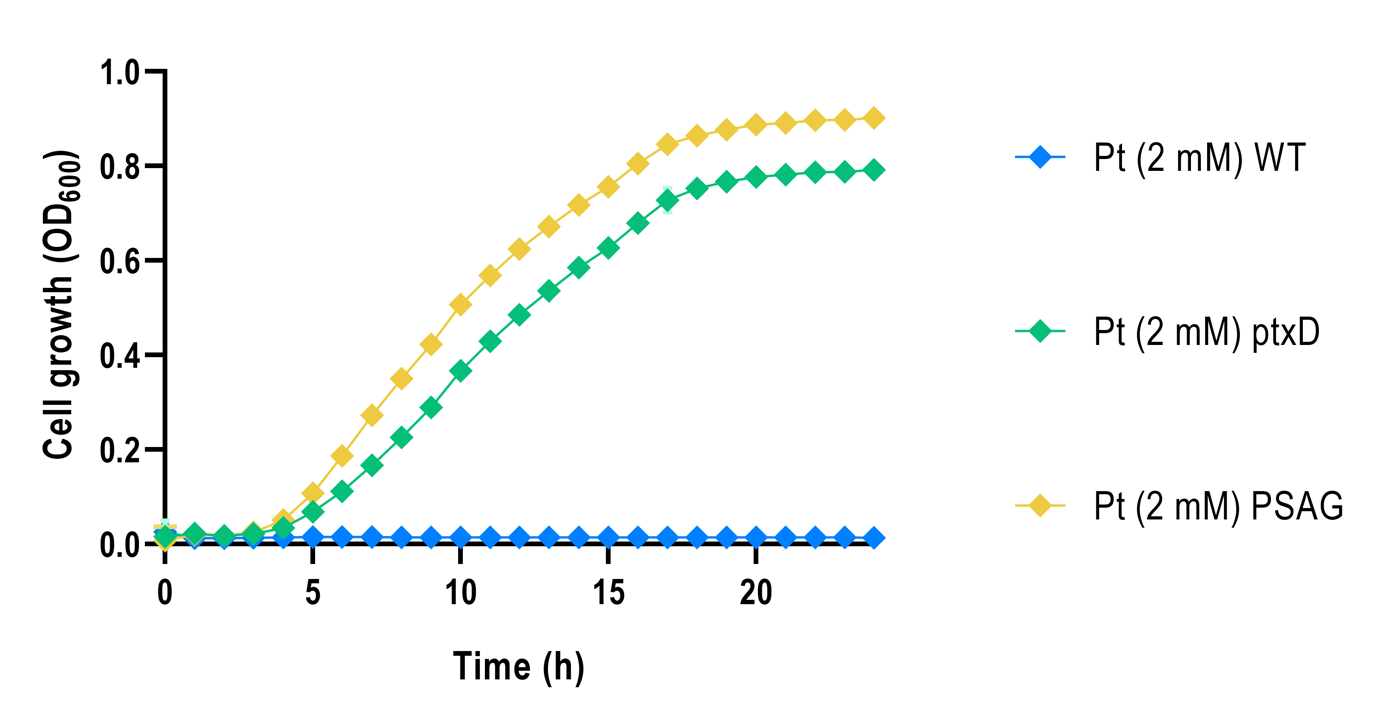
**

**Supplementary Figure 1.** PtxD only is required to grow on Pt. Growth of P. putida wild type (blue), PtxD-containing strain (green) and PSAG (yellow) in MOPS with different P sources. Growth was monitored by measuring OD_600_ in an ELx808 Absorbance Microplate Reader (BioTek Instruments, Inc., VT, U.S.). Error bars represent the standard deviation among biological duplicates and technical triplicates for each condition.

**Supplementary Figure S2. Evolution of the growth of *P. putida* in different P sources after addition of the PSAG assimilation genes and the removal of its Pi native transporters.**

**
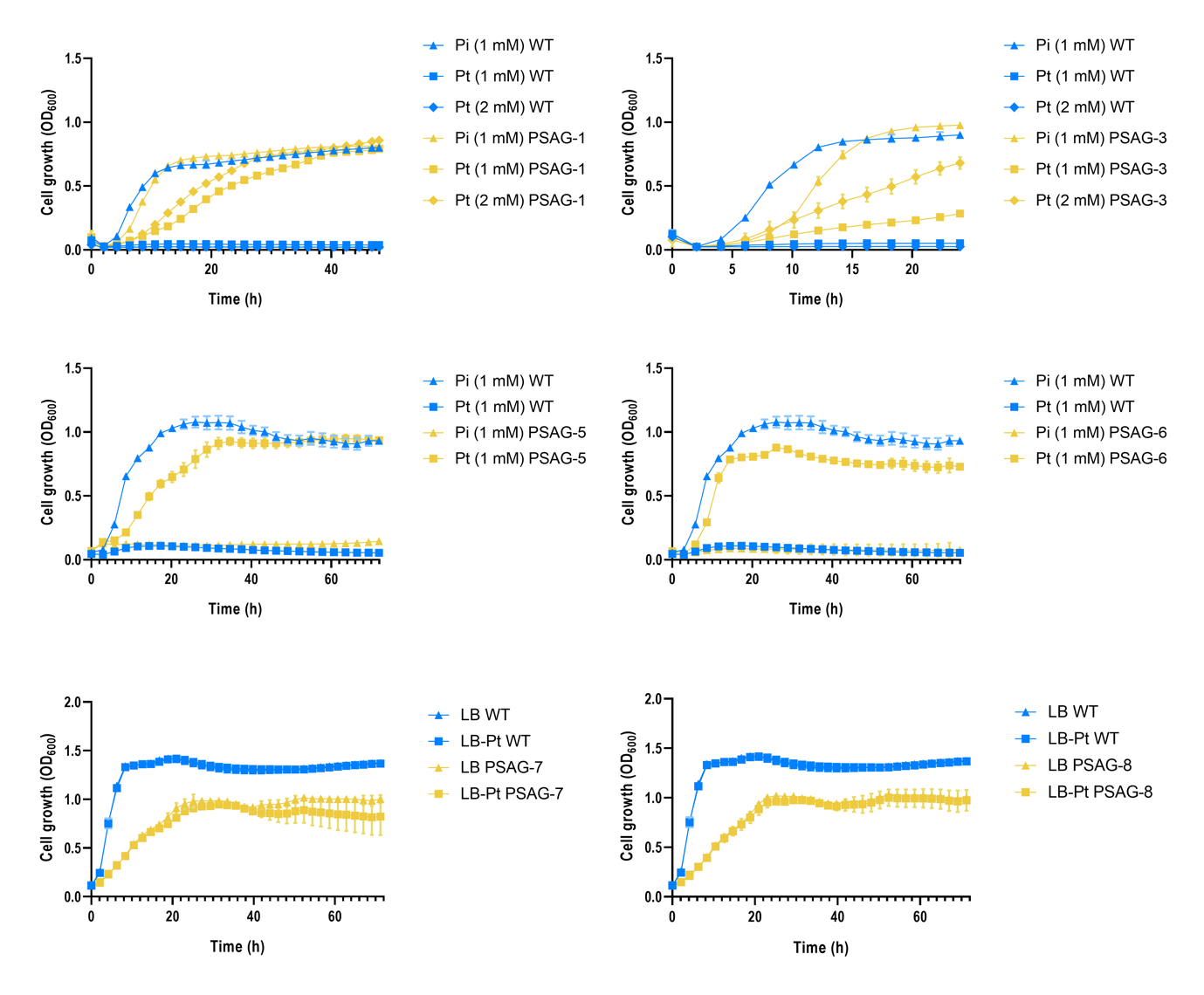
Supplementary Figure S5.2: Evolution of the growth of *P. putida* in different P sources after addition of the PSAG assimilation genes and the removal of its Pi native transporters.** (A) Growth of *P. putida* wild type (blue) and PSAG-1 (yellow) in MOPS with different P sources for 48 hours. (B) Growth of *P. putida* wild type (blue) and PSAG-3 (yellow) in MOPS with different P sources for 24 hours. (C) Growth of *P. putida* wild type (blue) and PSAG-5 (yellow) in MOPS with different P sources for 72 hours. (D) Growth of *P. putida* wild type (blue) and PSAG-6 (yellow) in MOPS with different P sources for 72 hours. (E) Growth of *P. putida* wild type (blue) and PSAG-7 (yellow) in LB with and without Pt for 72 hours. (F) Growth of *P. putida* wild type (blue) and PSAG-8 (yellow) in LB with and without Pt for 72 hours. Growth was monitored by measuring OD_600_ in an ELx808 Absorbance Microplate Reader (BioTek Instruments, Inc., VT, U.S.). Error bars represent the standard deviation among biological duplicates and technical triplicates for each condition.

**Supplementary Figure S3. PSAG-9 was the only bacteria that grew in MOPS-Pt under the non-sterile condition.**

**
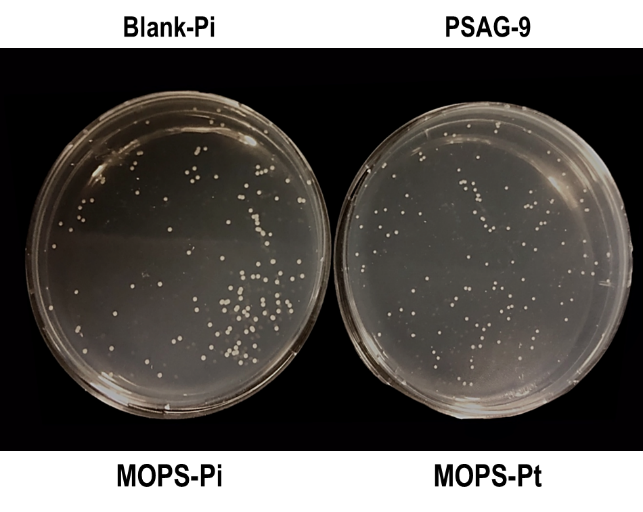
**

**Supplementary Figure S3.** The cultures were collected from the initially blank MOPS-Pi (left) and MOPS-Pt inoculated with PSAG-9 (right) and were plated onto MOPS - 50 mM Glucuse- 1 mM Pi and MOPS – 50 mM Glucose – 1mM Pt plates, respectively. Bacterial diversity was distinguished by colony morphology and verified by PCR. Picture was taken after 24 h.

**Supplementary Figure S4. PxtD has a NAD+/NADH conversion activity and wild type *P. putida***

**Supplementary Figure S4.** NADH production is detected in both wild type and PtxD-containing cell extracts. Wells were seeded with 0.3-0.45 mg/mL of total protein from clarified cell extracts. Values were normalized for total protein concentration in each extract. OD_340_ was measured for 90 min in 30-sec intervals. Error bars represent the standard deviation between biological triplicates and technical duplicates in each condition.

**demonstrates a weaker activity**

**Supplementary Table S1.**

Supplementary Table S1. Strains used in this study with their corresponding characteristics.

| **Organism** | **Strain** | **Characteristics** | **Experiment used** | **Source** |
| --- | --- | --- | --- | --- |
| *Escherichia coli* | DH5α | F^–^ *endA1* *glnV44* *thi-1* *recA1* *relA1* *gyrA96* *deoR* *nupG* *purB20* φ80d*lacZ*ΔM15 Δ(*lacZYA-argF*)U169, hsdR17(*r_K_*^–^*m_K_*^+^), λ^–^ |  | In-house strain |
| *Escherichia coli* | HB101 | F- Lambda- araC14 leuB6(Am) DE(gpt-proA)62 lacY1 glnX44(AS) galK2(Oc) recA13 rpsL20(strR) xylA5 mtl-1 thiE1 hsdS20(rB-, mB-) containing plasmid pRK600 for conjugation |  | In-house strain |
| *Pseudomonas putida* | KT2440 | *P. putida* KT2440 |  | In-house strain |
| *Escherichia coli* | DH5α | *E. coli* DH5α with the pSEVAb84 ptxD htxBCDE | Cloning purposes | This work |
| *Escherichia coli* | DH5α | *E. coli* DH5α with the pSEVAb22 cre + ptxD | Cloning purposes | This work |
| *Escherichia coli* | DH5α | *E. coli* DH5α with the pSEVAb22 cre + cassette ptxD htxBCDE | Cloning purposes | This work |
| *Pseudomonas putida* | KT2440 | *P. putida* KT2440 with landing pad lox71 + lox 66/2m in attTn7 locus | PSA strain construction | In-house strain |
| *Pseudomonas putida* | KT2439 | *P. putida* KT2440 with ptxD in the genomic attTn7 locus | PSA strain construction | This work |
| *Pseudomonas putida* | KT2440 | *P. putida* KT2440 with ptxD and htxBCDE in the genomic attTn7 locus --> *P. putida* PSAG | PSA strain construction | This work |
| *Escherichia coli* | DH5α | *E. coli* DH5α with the pGNW pitB KO | Cloning purposes | This work |
| *Escherichia coli* | DH5α | *E. coli* DH5α with the pGNW pitA KO | Cleavage essays and cloning purposes | This work |
| *Escherichia coli* | DH5α | *E. coli* DH5α with the pGNW pstSCAB KO | Cloning purposes | This work |
| *Escherichia coli* | DH5α | *E. coli* DH5α with the pGNW pstSCAB-II KO | Cloning purposes | This work |
| *Escherichia coli* | DH5α | *E. coli* DH5α with the pGNW phnCEptxBC KO | Cloning purposes | This work |
| *Escherichia coli* | DH5α | *E. coli* DH5α with the pGNW PP_3818 KO | Cloning purposes | This work |
| *Escherichia coli* | DH5α | *E. coli* DH5α with the pGNW yjbB KO | Cloning purposes | This work |
| *Escherichia coli* | DH5α | *E. coli* DH5α with the pGNW PP_2260/3 KO | Cloning purposes | This work |
| *Escherichia coli* | DH5α | *E. coli* DH5α with the pGNW phoBR KO | Cloning purposes | This work |
| *Pseudomonas putida* | PSAG1 | *P. putida* PSAG ΔpitB | PSA strain construction | This work |
| *Pseudomonas putida* | PSAG2 | *P. putida* PSAG ΔpitB ΔpitA | PSA strain construction | This work |
| *Pseudomonas putida* | PSAG3 | *P. putida* PSAG ΔpitB ΔpitA ΔpstSACB | PSA strain construction | This work |
| *Pseudomonas putida* | PSAG4 | *P. putida* PSAG ΔpitB ΔpitA ΔpstSACB ΔpstSCAB-II | PSA strain construction | This work |
| *Pseudomonas putida* | PSAG5.ph | *P. putida* PSAG ΔpitB ΔpitA ΔpstSACB ΔpstSCAB-II ΔphnCEptxBC | PSA strain construction | This work |
| *Pseudomonas putida* | PSAG5.pp | *P. putida* PSAG ΔpitB ΔpitA ΔpstSACB ΔpstSCAB-II ΔPP_3818 | PSA strain construction | This work |
| *Pseudomonas putida* | PSAG.6 | *P. putida* PSAG ΔpitB ΔpitA ΔpstSACB ΔpstSCAB-II ΔphnCEptxBC ΔPP_3818 | PSA strain construction | This work |
| *Pseudomonas putida* | PSAG7.y | *P. putida* PSAG ΔpitB ΔpitA ΔpstSACB ΔpstSCAB-II ΔphnCEptxBC ΔPP_3818 ΔyjbB | PSA strain construction | This work |
| *Pseudomonas putida* | PSAG7.g | *P. putida* PSAG ΔpitB ΔpitA ΔpstSACB ΔpstSCAB-II ΔphnCEptxBC ΔPP_3818 ΔPP_2260/3 | PSA strain construction | This work |
| *Pseudomonas putida* | PSAG7.p | *P. putida* PSAG ΔpitB ΔpitA ΔpstSACB ΔpstSCAB-II ΔphnCEptxBC ΔPP_3818 ΔphoBR | PSA strain construction | This work |
| *Pseudomonas putida* | PSAG8 | *P. putida* PSAG ΔpitB ΔpitA ΔpstSACB ΔpstSCAB-II ΔphnCEptxBC ΔPP_3818 ΔyjbB ΔPP_2260/3 | PSA strain construction | This work |
| *Pseudomonas putida* | PSAG9 | *P. putida* PSAG ΔpitB ΔpitA ΔpstSACB ΔpstSCAB-II ΔphnCEptxBC ΔPP_3818 ΔyjbB ΔPP_2260/3 ΔphoBR | PSA strain construction | This work |

**Supplementary Table S2.**

Supplementary Table S2. Plasmids used in this study with their corresponding characteristics.

| **Plasmid ID** | **Description** | **Experiment used** | **Source** |
| --- | --- | --- | --- |
| **pSTV28 htxBCDE** | Plasmid STV29 carrying phosphite transporter from *Pseudomonas stutzeri* WM88. Chloramphenicol resistance. | PSA strain construction | Kind gift from Hirota's lab |
| **pSTV28 ptxD** | Plasmid STV29 carrying the phosphite dehydrogenase gene from *Ralstonia sp*. 4506. Chloramphenicol resistance. | PSA strain construction | Kind gift from Hirota's lab |
| **pSEVAb84 ptxD htxBCDE** | Plasmid SEVAb84 carrying the PSAG expression cassette (ptxD + htxBCDE). Apramycin resistance. | PSA strain construction | This study |
| **pGNW landing pad lox71 + lox66/2m KI in attTn7 locus** | Plasmid for genomic integration in the locus attTn7 of the *P. putida* genome of a landing pad with lox71 and lox66/2m sites. Kanamycin resistance. | PSA strain construction | In-house |
| **pQURE6** | Plasmid carrying Gentamicin resistance gene, xylS/pM regulator/promoter system controlling plasmid replication and I-SceI expression. | Through | Kind gift from Nikel's lab |
| **pSEVAb22 cre + ptxD** | Plasmid SEVAb22 for cre integration of ptxD in landing pad attTn7. Kanamycin resistance. | PSA strain construction | This study |
| **pSEVAb22 cre + cassette ptxD htxBCDE** | Plasmid SEVAb22 for cre integration of cassette ptxD + htxBCDE in landing pad attTn7. Kanamycin resistance. | PSA strain construction | This study |
| **pGNW** | Plasmid carrying kanamycin resistance gene, R6K origin of replication, GFP gene, multiple cloning site, 2 I-SceI recognition sites flanking the multiple cloning site. | Through | In-house |
| **pGNW pitB KO** | Plasmid for genomic deletion of pitB gene in *P. putida*. Kanamycin resistance. | PSA strain construction | This study |
| **pGNW pitA KO** | Plasmid for genomic deletion of pitA gene in *P. putida*. Kanamycin resistance. | PSA strain construction | This study |
| **pGNW pstSCAB KO** | Plasmid for genomic deletion of pstSCAB genes in *P. putida*. Kanamycin resistance. | PSA strain construction | This study |
| **pGNW pstSCAB-II KO** | Plasmid for genomic deletion of pstSCAB-II genes in *P. putida*. Kanamycin resistance. | PSA strain construction | In-house |
| **pGNW pnhCEptxBC KO** | Plasmid for genomic deletion of phnCEptxBC genes in *P. putida*. Kanamycin resistance. | PSA strain construction | This study |
| **pGNW PP_3818 KO** | Plasmid for genomic deletion of PP_3818 gene in *P. putida*. Kanamycin resistance. | PSA strain construction | This study |
| **pGNW yjbB KO** | Plasmid for genomic deletion of ynbB gene in *P. putida*. Kanamycin resistance. | PSA strain construction | This study |
| **pGNW PP_2260/3 KO** | Plasmid for genomic deletion of PP_2260/3 genes in *P. putida*. Kanamycin resistance. | PSA strain construction | This study |
| **pGNW phoBR KO** | Plasmid for genomic deletion of phoBR genes in *P. putida*. Kanamycin resistance. | PSA strain construction | This study |

**Supplementary Table S3.**

Supplementary Table S3. Oligonucleotides used in this study with their corresponding characteristics.

| **PCR amplification primers** | | |
| --- | --- | --- |
| **Name** | **Sequence (5'→ 3')** | **Description** |
| **C1 pSEVAb84 BB FW** | AGGTCTCTTACTAGTAGCGGCCGCTGC | Amplification of the backbone pSEVAb84 |
| **C2 pSEVAb84 BB RV** | GGTCTCTCTCTAGAAGCGGCCGCGAAT |  |
| **C3 ptxD FW** | AGGTCTCTGAATCCCTGTTGACAATTAATCATCGGCTC | Amplification of the ptxD from *Ralstonia sp*. 4506 From pSTV28 |
| **C4 ptxD RV** | AGGTCTCTCGACGACAGGAAGAGTTTGTAGAAACG |  |
| **C5 htxBCDE FW** | AGGTCTCTGTCGTAGCTCACTCATTAGGCACCCC | Amplification of the htxBCDE operon from *P. stutzeri* WM88 from pSTV28 |
| **C6 htxBCDE BsaI RV** | TCGACGGTTTCCGTCGTGGCTTCCATCTTCAGCGTGTCCAGATTCATCTTCTGCTCGACGCGCTCCTGGGTTTCGATGTGCGA |  |
| **C7 htxBCDE BsaI FW** | AGCCACGACGGAAACCGTCGAAGTGCTGGTCAAGCCGGTCGGCTATGTCTGGACGGTTTTCATCAAGATGATCGAAACCCTGGAGAT |  |
| **C8 htxBCDE RV** | AGGTCTCTACTGTCAGATCAGCTTGGCGCG |  |
| **C9 crelox BB FW** | AGGTCTCTCTAGTACCGTTCGTATATGGTTTCTTAT | Amplification of the backbone of pSEVAb22 cre lox for genome integration |
| **C10 crelox BB RV** | AGGTCTCTAGTGTACCGTTCGTATAATGTATGC |  |
| **C11 PSAG for landing pad FW** | AGGTCTCTCACTCCCTGTTGACAATTAATCATCGGCTCG | Amplification of ptxD and ptxD+htxBCDE for cloning into pSEVAb22 cre lox for genome integration |
| **C12 ptxD for landing pad RV** | AGGTCTCTCTAGGCCTGGGGTGCCTAATGAGT |  |
| **C13 PSAG for landing pad RV** | AGGTCTCTCTAGTCAGATCAGCTTGGCGCGG |  |
| **P1 pGNW BB FW** | AGGTCTCTTCGACCTGCAGGCATGC | Amplification of pGNW BB |
| **P2 pGNW BB RV** | AGGTCTCTCGGGTACCGAGCTCGAATTC |  |
| **P3 HR1 pitB FW** | AGGTCTCTCCCGAACTGCGCGCGGAAC | Amplification of homology region 1 for pitB deletion |
| **P4 HR1 pitB RV** | AGGTCTCTGAAGGTTGTCTGGCGGG |  |
| **P5 HR2 pitB FW** | AGGTCTCTCTTCGTGACACCGCAATGAATGAA | Amplification of homology region 2 for pitB deletion |
| **P6 HR2 pitB RV** | AGGTCTCTTCGAGGTGGGTACGTTGCAAG |  |
| **P7 HR1 pitA FW** | AGGTCTCTCCCGGCGTGTCGCCTTCTATCACC | Amplification of homology region 1 for pitA deletion |
| **P8 HR1 pitA RV** | AGGTCTCTCATGGGGGCTCCGGTT |  |
| **P9 HR2 pitA FW** | AGGTCTCTCATGTGAACCCGTACGCCGC | Amplification of homology region 2 for pitA deletion |
| **P10 HR2 pitA RV** | AGGTCTCTTCGACCCCGAGTTCATCTCGCT |  |
| **P11 HR1 pstSCAB FW** | AGGTCTCTCCCGTATTGCATGAGTCATCTTGATGCTTTCA | Amplification of homology region 1 for pstSCAB deletion |
| **P12 HR1 pstSCAB BsaI RV** | AGGTCTCTATCACCGGCATCCC |  |
| **P13 HR1 pstSCAB BsaI FW** | AGGTCTCTTGATACAGGACTCGCCGG |  |
| **P14 HR1 pstSCAB RV** | AGGTCTCTGTGATGGCCGGCAC |  |
| **P15 HR2 pstSCAB FW** | AGGTCTCTTCACACCATGCAAGCTCAAGGTGG | Amplification of homology region 2 for pstSCAB deletion |
| **P16 HR2 pstSCAB RV** | AGGTCTCTTCGAGGATCCGTACATACACACCC |  |
| **P17 HR1 pstSCAB-II FW** | AGGTCTCTCCCGTCCCGCTGTATGCCGTGAC | Amplification of homology region 1 for pstSCAB-II deletion |
| **P18 HR1 pstSCAB-II RV** | AGGTCTCTACGCGCCTTGCTCCTAGCAGGAATATTGG |  |
| **P19 HR2 pstSCAB-II FW** | AGGTCTCTGCGTGCGCCGCTCGCGAAA | Amplification of homology region 2 for pstSCAB-II deletion |
| **P20 HR2 pstSCAB-II RV** | AGGTCTCTTCGAAATGTGGCGAACCTCGACATAACCA |  |
| **P21 HR1 phnCEptxBC FW** | AGGTCTCTCCCGTTTACTGACCACCCCGGT | Amplification of homology region 1 for phnCEptxBC deletion |
| **P22 HR1 phnCEptxBC BsaI RV** | AGGTCTCTACTTCTTCATGCCGATCGTCGATG |  |
| **P23 HR1 phnCEptxBC BsaI FW** | AGGTCTCTAAGTCGGTGGTCGAAACCAC |  |
| **P24 HR1 phnCEptxBC RV** | AGGTCTCTGGGTGTTCTCCGGTTCAATAGC |  |
| **P25 HR2 phnCEptxBC FW** | AGGTCTCTACCCCGGAAAGCCCGGGCTG | Amplification of homology region 2 for phnCEptxBC deletion |
| **P26 HR2 phnCEptxBC RV** | AGGTCTCTTCGATCAAGGCCACCCTGCTG |  |
| **P27 HR1 PP_3818 FW** | AGGTCTCTCCCGGCCATTCGCTTGACCC | Amplification of homology region 1 for PP_3818 deletion |
| **P28 HR1 PP_3818 RV** | AGGTCTCTGCCGGGTGACCTGTG |  |
| **P29 HR2 PP_3818 FW** | AGGTCTCTCGGCTCATCAGCGGGTGACGG | Amplification of homology region 2 for PP_3818 deletion |
| **P30 HR2 PP_3818 BsaI RV** | AGGTCTCTGTGGTCGATGCCAAGACCGACAAGGTGC |  |
| **P31 HR2PP_3818 BsaI FW** | AGGTCTCTCCACCAACTTCATCAAGGTCTTTTCC |  |
| **P32 HR2 PP_3818 RV** | AGGTCTCTTCGAATGAGCAGTACCAGACCACC |  |
| **P33 HR1 yjbB FW** | AGGTCTCTCCCGCGCTGAAGATGCTCGCGCTG | Amplification of homology region 1 for yjbB deletion |
| **P34 HR1 yjbB RV** | AGGTCTCTGGCGAAGGCCCCCTG |  |
| **P35 HR2 yjbB FW** | AGGTCTCTCGCCTTTCTCCTAGCTGAGGACCC | Amplification of homology region 2 for yjbB deletion |
| **P36 HR2 yjbB RV** | AGGTCTCTTCGATGCAGATGGCCATAGTGG |  |
| **P37 HR1 PP_2260/3 FW** | AGGTCTCTCCCGCGATGAAACGCCGCGAGC | Amplification of homology region 1 for PP_2260/3 deletion |
| **P38 HR1 PP_2260/3 RV** | AGGTCTCTGGCGTGGCCTTTTTGTGATTG |  |
| **P39 HR2 PP_2260/3 FW** | AGGTCTCTCGCCGGAGGGCAGCGCATGGA | Amplification of homology region 2 for PP_2260/3 deletion |
| **P40 HR2 PP_2260/3 RV** | AGGTCTCTTCGACACTGCAATTCGGCCAACTGCTG |  |
| **P41 HR1 phoBR FW** | AGGTCTCTCCCGTTGGCAACGTGCCAGAC | Amplification of homology region 1 for phoBR deletion |
| **P42 HR1 phoBR RV** | AGGTCTCTTAGATAACGGAATTATTGCAGCTGTG |  |
| **P43 HR2 phoBR FW** | AGGTCTCTTCTAGATTGAGGCTGCGGC | Amplification of homology region 2 for phoBR deletion |
| **P44 HR2 phoBR RV** | AGGTCTCTTCGAGCTGTCCACGCCGAGATA |  |
| **V1 attTn7 FW** | AGCGCGAATGTAATTAAGCG | Verification primers to check attTn7 integration |
| **V2 attTn7 RV** | TCAGCACTGATTGACTCTTG |  |
| **V3 pitB FW** | CCAAGGAGCAGAAGTCGCTG | Verification primers to check pitB deletion |
| **V4 pitB RV** | TTTATCAAGCCGTTGCGGCC |  |
| **V5 pitA FW** | GTAAAGGTGTGGATCCACTATGCC | Verification primers to check pitA deletion |
| **V6 pitA RV** | CGAACTCAGTGCTTTCCCCATTAC |  |
| **V7 pstSCAB FW** | GGTTTGCAGCCTTGGAGAA | Verification primers to check pstSCAB deletion |
| **V8 pstSCAB RV** | CGGCACGCATCAGCAAATA |  |
| **V9 pstSCAB-II FW** | GCGGTCTTGTATTCGCGGT | Verification primers to check pstSCAB-II deletion |
| **V10 pstSCAB-II RV** | CCTGGCCGACCGCTATG |  |
| **V11 phnCEptxBC FW** | TGGCCTTCTTTTCGGCAGT | Verification primers to check phnCEptxBC deletion |
| **V12 phnCEptxBC RV** | AGTTTTCGCAACAGCAGCG |  |
| **V13 yjbB FW** | GCCCAAAGGCGCAGGTGG | Verification primers to check yjbB deletion |
| **V14 yjbB RV** | GATAGGCCGGAAGCAGGCGG |  |
| **V15 PP_2260/3 FW** | GAGCGCCCCGACCAGCT | Verification primers to check PP_2260/3 deletion |
| **V16 PP_2260/3 RV** | GATGTTCTTGTCCGACTGCATCTGCG |  |
| **V17 phoBR FW** | GGTCAACGCGCAAGCTGGACC | Verification primers to check phoBR deletion |
| **V18 phoBR RV** | GAACAGGTAAAGCGGCACGGCG |  |
| **V19 86** | CTGGATTCTCACCAATAAAAAACG | Verification of any cargo of a SEVA plasmid |
| **V20 87** | TCTAGGGCGGCGGAT |  |

**Supplementary Table S4.**

Supplementary Table S4. Additional DNA sequences used in this study with their corresponding characteristics.

| **Name** | **Sequence (5'→ 3')** | **Source** |
| --- | --- | --- |
| ***ptxD Ralstonia sp*. 4506** | atgaagcccaaagtcgtcctcacccactgggtgcacccggaaatcatcgaattgttgtccgctagcgccgatgttatccccaacaccacacgggaaaccttgccgcgttctgaggtaattgcgcgagccaaagatgcggatgcactcatggctttcatgccggacagcatcgacagcgcgtttctcgaggaatgtccaaagctgcgtgtcatcggcgccgcgcttaaaggctatgataacttcgatgtcaacgcctgcacacgccacggtgtatggcttacgattgtgccggatttgcttacgatcccgaccgctgaactgactatcggccttcttctcggtttgacaaggcatatgctggaaggcgataggcaaatccgtagcggacacttccaaggctggcggccgacactatatggctctggtttgacaggaaaaacgcttggcatcattggtatgggggcggtcggccgtgcaatcgcccagcgcttggctggctttgaaatgaatctcttgtattgcgatccgattccgctcaatgccgaacaagaaaaggcttggcacgtacagcgcgtcacgctcgatgaactgctcgaaaaatgtgattatgtcgtgccgatggttccgatggccgcagagacactgcatctgatcgatgccaccgcgttggccaagatgaaaaccggtagctacctgatcaatgcatgtcgcggctcggtcgtggatgagaatgcggtgatagcagcactggcgtctggaaaactagctggatatgcagccgatgtcttcgagatggaagaatggatacgcgctgatcgcccgcaggctatccccaaggcgctgctcgacaatacggcacaaacgttttttacgccgcatttgggatcggcggtcaaggaagttcggcttgaaatcgagcggcaggcagcgatgaacatcatccaggcactcgctggtgaaaaaccgatgggcgcgattaatcagccgtatccgggagtaaaggcggcgtga | Hirota *et al*., 2012 |
| ***htxBCDE* *P. stutzeri* WM88** | atgcaagtttttactctgttttcgaaattcaagaaggcgttaacgcgcgccattcttgcctttatcgccacaatcatagtgtgcacacccgcgcaggcagctgaggttgtcaatggtaaacttcacctgcgttttgcaattgcgccgatgcgtccaacgcctagccagaccatcaaagagtttgagccgatattcaagtatctcgccgaccagctcggcgcgacctatgaaatcgtctccccggaaagctgggcggcaatatctgtggcaatgacaaatggccatgtcgatgtgggctggctcggaccctggggctatgtcttgtcgaataaaaaggccggcaccgaagtgcttgcaacggtcaagtaccgcggggagccgttctacaaagccctcattgtcggtcgcgccgatctgccgatcaaaaaatggcccgaggacgcgaagggtttgaagctgtcactcagtgatcagggcaacacttctggctggctcatcccgatggcgtacttcaagagcatcggcatcgaccctgcgagctattttgaatatcgtgaaggtgccacgtttggccagaacgaatcacagattcagcacggactgatcgacctcggatccgatatggatcggggccggaacgggatgatcgaagcgggtcaaatcgatccttcgaagtccaagatcgtgtgggaatccagcaagctgccgaacgacgcgatatccgtgccgaaggattttgatcctgctctgaaagcgcgcatcacggaaatactgacgtccttgtccgaagagaaagcacagtcgctgatgggctcgggctataacggcttcgtgaaggcaaagcacagcgattacaaggtaatcgaagacgccggccgcatcctgggaaaactgtaaagcacgaggggtccgttcttggatgagggcagcggacgacaaggtggactgacgcacgccagctccttgtctccgctgcacgaacatacgggcgcgcatcgcaataccacagaggatgaaccaatgaatcagcgaatcgaagaagtcatgctggctaatgtcaagagggacgtagccaggagaaagcggcattttgcaacgtcggtcgtagtactcagtttgctggcagtggcctggtacgtgtgtcagatagaattccagaagctaggcgccggtttaccgagactatggtcattcgtcgtgcagatgtttccacccgacctgagcgacctggacgtcattctaaaaggggctggcgagacgctcgccatggcgacgattggcacgatattcgccacaatcattgcatttccgctggcactcatggctgcgcgtaatacctgtccgaacaagtggacctatcgggtatcccgcgccatcctgaacgccagccgcggcacggagacatttgtctatgcacttgtatttgtagcagcagtgggcttcggtccgttctccggcgtactggccattactttccacatggtaggggcaatcggcaaaatgtttgctgaagccatcgagcccgttgaccaagggccgttggatgcgctcgccttgaccggtgccagcagggcaaagattatccgctacggtctgatcccggatgttatgccgcacctgatcgcgagcgttctatacatttgggaattcagtgtcagaacgtccacagtactgggcatcgtaggcgcaggtggaattgggcagaccctgaaagatactgtggacttgttggaattcaacaagatgattacggtactggcggttgtattgctgatggtgtcggcaatcgatttcatcagtgaccggctcaggtacttgatattggacacaaaacgcgagggattcgaaactctccctgcgaataactgattgcttcacgtattactggaagggaggttcgcaatgaaagatgtagcgttgcagttaaagaatgtcggtaagtcatacggcaataaagttgtcctggaatcgattgacttcgaagtacgtcacggctcaatggttgccttgctcggcacaagcggggcagggaagtcgacgcttttccgatgtctcactggccttgagccgattgactccggttctatcgtggcgctcggagaatccatacatgaactgtctccggcgcgtctgcgggcagtacgtggccagatcgggttcgtgttccaacaactgcacctggtgaaaaggttctcagcactcgagaatgtattgggtgcgcgtctggcagagatgcccatttggcgcgtcacattgaaaagcttcagccgggctgacaaagtgctcgcgttcgaatgtctggaccgggtcggcatgctcgattatgcaaacacgcctacgcaactgctgtcaggcggtcagcaacagcgtattgcgatagcgcgagccttggcgcagaagcccaagattattattgcggacgaacccgtctccagcctcgatccgctgacggcgcgctcggttctgcaaacgctgaaagccgcggctacagatcttaatgtcgcggtcctgtgcagcctgcaccaggtagacctggcccgtgagtttggcgaccgcatcgtgggcatgcgcgacggacgtgtcgttttcgacggcacgccagcggaattcaccgacgagcgcgtgcatgcgctttaccagggtgcccgctgggaagatgcaccagcggccgagagcgacgcgcagcactcggtggccggtctggctgtggcatgaggggcgaagcgatgaccacatccacacgccccatacccgtgccgccccagggcaccgcactgcactggcacctgagcgcgccctacaacgccaaacatctgctggtgctgatcgccgtcatggtgctgttgttcgtgaccggacaacgcaccgaaatggaccgcatggtggccatgacggcacaggccgtggccaagaccgtgggcctggctgacgattcacaagtcgcgcgcggcttgtcgcgcgtcggtcaagccatgtggccacccgccatcgcagaaaccgaagaggtgggccggattcaggacctggatcgccagaagctgcccctgttctcgcacatcGAAacccaggagcgcgtcgagcagaagatgaatctggacacgctgaagatggaagccacgacggaaaccgtcgaagtgctggtcaagccggtcggctatgtctggacggttttcatcaagatgatcGAAaccctggagattgcgctgtggggcacgatcctgtcggtgctggtgtcgattcccctggcgtatttcgcggcccgcaactactagccccaaccgttttacctacaccgctgcccgcggcaccatcagtctgctgcgttcagcgccggaactcatcgtcgctttgttcctggtgctggcctacggctttggccccatcgctggcgtgctggcgctgggcctgcatgcggccggcttcctgggcaagttctacgccgaggacatcgagaacgccgacaagaagccgcaagaggcgctggaggccatcggcgcgggcaagctcaagacgctgtggtacggcgtcatcccccaggtcttgccgcaatacatcgcctacaccgcctacatcctggaccgcaacctgcgcatggccaccgtcatcggtctggtgggcgcgggcggcatcggccaggaactcaagggccgttttgacatgttccagtacggccatgtcatgaccatcctgatcgcgatcttcgtctttgtgttcgtgctggaccagttgcaggcgcgcatccgcgccaagctgatctga | Hirota's lab |

1. Department of Life Sciences, Imperial College London, Exhibition Road, South Kensington, London SW72BX United Kingdom [↑](#footnote-ref-1)
2. The Novo Nordisk Foundation Center for Biosustainability, Technical University of Denmark, Kgs. Lyngby, 2800 Denmark [↑](#footnote-ref-2)
